# Perioperative Care Pathways in Low- and Lower-Middle-Income Countries: Systematic Review and Narrative Synthesis

**DOI:** 10.1007/s00268-022-06621-x

**Published:** 2022-06-22

**Authors:** Jignesh Patel, Timo Tolppa, Bruce M. Biccard, Brigitta Fazzini, Rashan Haniffa, Debora Marletta, Ramani Moonesinghe, Rupert Pearse, Sutharshan Vengadasalam, Timothy J. Stephens, Cecilia Vindrola-Padros

**Affiliations:** 1 Division of Surgery and Interventional Science, Centre for Perioperative Medicine, University College London, London, UK; 2 Network for Improving Critical Care Systems and Training, YMBA Building, Colombo, 08 Sri Lanka; 3 Mahidol Oxford Tropical Medicine Research Unit, Bangkok, 10400 Thailand; 4 Department of Anesthesia and Perioperative Medicine, Groote Schuur Hospital and University of Cape Town, Cape Town, South Africa; 5 Adult Critical Care Unit, The Royal London Hospital, Barts Health NHS Trust, Whitechapel, London, E1 1FR UK; 6 Library Services, University College London, London, UK; 7 Critical Care and Perioperative Medicine Research Group, William Harvey Research Institute, c/o ACCU Research Team, Royal London Hospital, Queen Mary University of London, London, E1 1BB UK; 8 Department of Surgery, Jaffna Teaching Hospital, Jaffna, 40000 Sri Lanka; 9 Division of Surgery, Department of Targeted Intervention, University College London, London, UK

## Abstract

**Background:**

Safe and effective care for surgical patients requires high-quality perioperative care. In high-income countries (HICs), care pathways have been shown to be effective in standardizing clinical practice to optimize patient outcomes. Little is known about their use in low- and middle-income countries (LMICs) where perioperative mortality is substantially higher.

**Methods:**

Systematic review and narrative synthesis to identify and describe studies in peer-reviewed journals on the implementation or evaluation of perioperative care pathways in LMICs. Searches were conducted in MEDLINE, EMBASE, CINAHL Plus, WHO Global Index, Web of Science, Scopus, Global Health and SciELO alongside citation searching. Descriptive statistics, taxonomy classifications and framework analyses were used to summarize the setting, outcome measures, implementation strategies, and facilitators and barriers to implementation.

**Results:**

Twenty-seven studies were included. The majority of pathways were set in tertiary hospitals in lower-middle-income countries and were focused on elective surgery. Only six studies were assessed as high quality. Most pathways were adapted from international guidance and had been implemented in a single hospital. The most commonly reported barriers to implementation were cost of interventions and lack of available resources.

**Conclusions:**

Studies from a geographically diverse set of low and lower-middle-income countries demonstrate increasing use of perioperative pathways adapted to resource-poor settings, though there is sparsity of literature from low-income countries, first-level hospitals and emergency surgery. As in HICs, addressing patient and clinician beliefs is a major challenge in improving care. Context-relevant and patient-centered research, including qualitative and implementation studies, would make a valuable contribution to existing knowledge.

**Supplementary Information:**

The online version contains supplementary material available at 10.1007/s00268-022-06621-x.

## Introduction

Improving access to surgical care remains a global priority due to persisting inequities and the considerable burden of surgical conditions. An estimated nine in ten people who live in low- and middle-income countries (LMIC) are unable to access safe affordable surgical care, leaving an unmet need for 143 million procedures to address avoidable surgical mortality and morbidity [1–3]. In addition to expanding surgical volume, strategies are also required to improve quality of surgical care. Mortality after surgery in LMICs is much higher compared to high-income countries (HICs) and is the third leading cause of global deaths according to some estimates [4–6]. Transnational research suggests that there may be inefficiencies throughout the perioperative care continuum, which encompasses all health system activities before, during and after surgery [7], which contribute to poor outcomes [4, 8]. Therefore, improvements in perioperative care are required to realize the aspiration of providing access to safe surgical care worldwide.

Care pathways are one way of achieving high quality perioperative care as they are multidisciplinary plans incorporating the best available evidence to organize clinical practice, optimize patient outcomes and maximize clinical efficiency [5, 9]. In HICs, implementation of care pathways has reduced length of hospital stay without increasing readmission rates [10]. However, little is known about the use of perioperative pathways in LMICs where more efficient use of limited resources is particularly relevant. This lack of context-specific knowledge is problematic for those in LMICs seeking to implement care pathways, as understanding context is key for those attempting to influence change [11, 12]. Health illiteracy, absence of equipment, a limited workforce and high healthcare costs are some contextual factors contributing to poor perioperative care, particularly in LMICs [13], which may impact the design and implementation of perioperative pathways.

The aim of this systematic review and narrative evidence synthesis was to identify and describe the body of literature regarding the implementation and evaluation of perioperative care pathways in LMICs. Our objective was to better understand the design, components, outcome measures and implementation strategies of pathways as well as implementation barriers and facilitators.

## Methods

We conducted a systematic review and narrative evidence synthesis according to the Preferred Reporting Items for Systematic Reviews and Meta-Analyses (PRISMA) statement [14]. The study protocol was registered with the international prospective register of systematic reviews (PROSPERO CRD42020172978) and reported in accordance with PRISMA and Synthesis Without Meta-Analysis guidelines (Online Resource 1) [14, 15].

### Search strategy

Searches were conducted on July 5, 2020 in MEDLINE (Ovid), EMBASE (Ovid), CINAHL Plus (Ebscohost), WHO Global Index, Web of Science (Core), Scopus, Global Health (Ovid) and SciELO electronic databases (Online Resource 2), supplemented by browsing reference lists for additional studies. The search strategy was developed in consultation with an experienced researcher (CV) and university librarian (DM). Search results were exported into the EndNote (Clarivate, USA) reference manager to remove duplicates.

### Selection of sources of evidence

Two researchers (JP, TT) independently screened titles, abstracts and full-text records, and included peer-reviewed articles written in English that described the implementation or evaluation of a perioperative care pathway in a LMIC involving patients of any age undergoing surgery. After an initial search, we found that a large proportion of eligible articles were from upper-middle-income countries (UMICs). We were concerned that such a disproportionate sample from the wealthiest LMIC settings would be poorly generalizable across resource-poor hospitals in LMICs. As such and in view of the resources available for this review, we prospectively excluded studies from UMICs and focused on low and low-middle income countries. Studies from UMICs will be reviewed separately (PROSPERO CRD42022324301).

Surgery was defined as a procedure taking place under the care of an anesthetist with a surgeon. A pathway was considered ‘perioperative’ if it concerned a journey through any combination of pre-, intra-, or postoperative phases. LMICs included upper-middle, lower-middle and low-income countries as per the World Bank [16]. A care pathway was defined as a structured multidisciplinary plan of care meeting at least three of the following criteria [5]:

1. Channels the translation of guidelines or evidence into local structures.
2. Details steps in a course of treatment or care in a plan, pathway, algorithm, guideline, protocol or other inventory of actions.
3. Has timeframes of criteria-based progression.
4. Aims to standardize care for a specific clinical problem, procedure or episode of care.

Conference abstracts, narrative reviews, letters, case reports and simulated evaluations were excluded. No exclusions were made based on comparators, outcomes or date. Decisions were recorded using the Rayyan QCRI web application [17] and discrepancies resolved by consensus or, failing that, a third researcher (TS).

### Data charting and synthesis of results

A data charting form was created, piloted on a random sample of 4 articles and modified accordingly before extracting the following: author, country, year of publication, aim, design, number of patients, type of institution, specialty and acuity of surgery, scale of implementation, components and design of pathways, implementation strategies, comparators, and outcome measures. Facilitators and barriers to pathway implementation were also sought. Quality assessments were carried out independently by two researchers using the Mixed Methods Appraisal Tool (MMAT) for descriptive statistics of included studies [18]. Disagreements were resolved by discussion.

We used descriptive statistics, taxonomy classifications and frameworks to summarize data. Institutions, in which care pathways were set, were categorized into first, second and third-level hospitals (Online Resource 3) [19]. We described the design of perioperative care pathways as adopted (used a previously developed pathway), adapted (modified a previously developed pathway) or designed de novo [20]. The scale of pathway implementation was denoted as either within a single clinical team (surgeon and associated perioperative team), hospital-wide, national or international. Pathway implementation strategies were categorized according to the Expert Recommendations for Implementing Change (ERIC) taxonomy [21, 22], outcome measures were categorized using the COMET (Core Outcome Measures in Effectiveness Trials) Initiative taxonomy [23], and facilitators and barriers to pathway implementation were aggregated using the Consolidated Framework for Implementation Research (CFIR) [24].

## Results

**Fig. 1:**
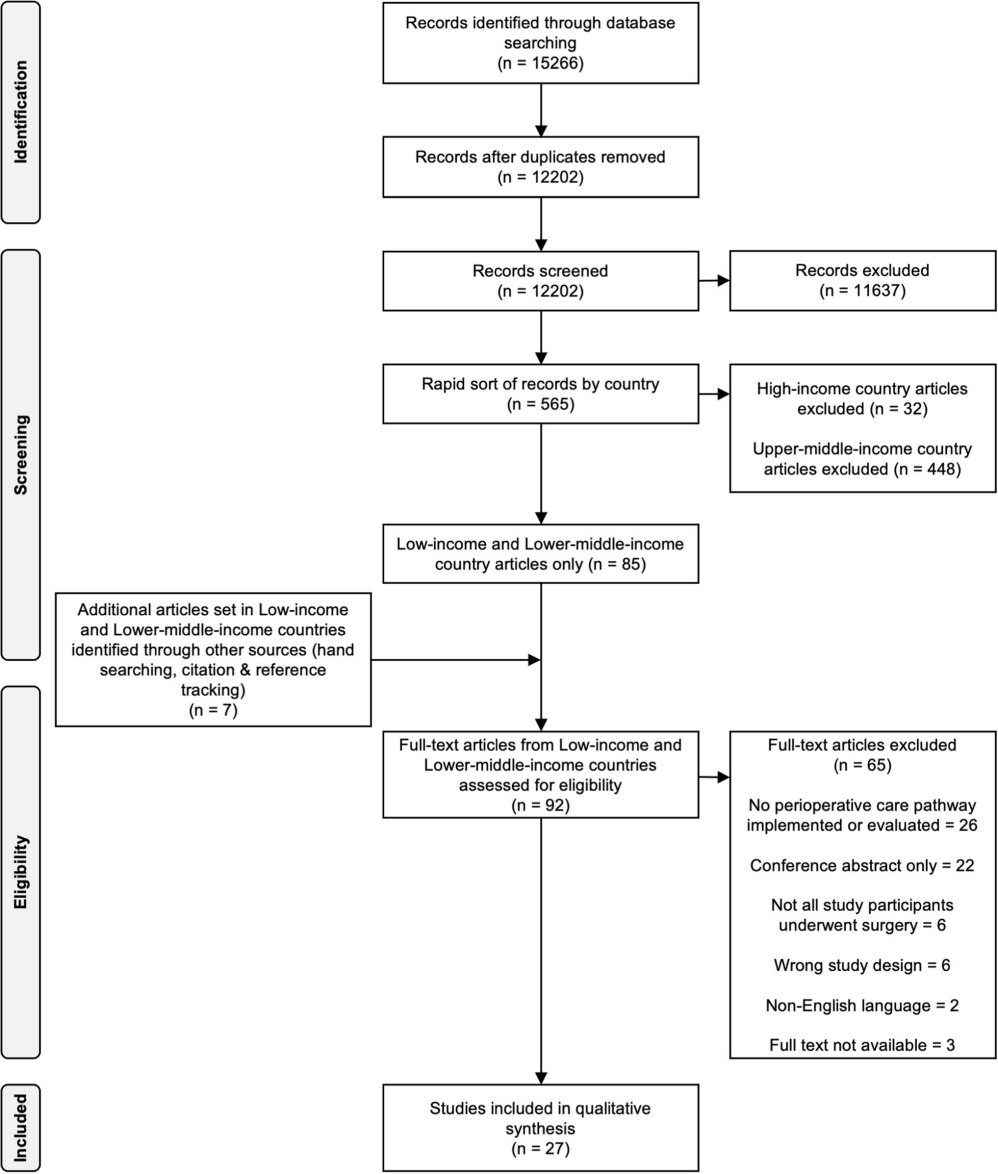
PRISMA diagram showing selection of articles for review

The flow diagram of study selection is shown in Fig. 1. The initial literature search identified 15,266 articles. We removed 3064 duplicates and excluded 11,637 articles after screening titles and abstracts. Thirty-two articles from HICs and 448 from UMICs were excluded following a rapid sort by country. Full-text records of 85 articles and 7 additional papers identified through citation tracking were reviewed. In instances where full-text articles were not available, attempts were made to directly contact the author. A total of 27 articles met the inclusion criteria.

### Characteristics of sources of evidence

Characteristics of included studies are summarized in Table 1. Over half (*n* = 15) were published in the last 5 years (2016–2020), and the earliest article is from the year 2000. All studies were conducted in a single country and were from seven different countries: Bangladesh, Egypt, India, Nepal, Pakistan, Uganda and Ukraine. Twenty-four studies (89%) were from lower-middle-income, and 3 (11%) from low-income countries. Just over half (*n* = 15, 56%) were from India.

**Table 1:** Characteristics of included studies and care pathways

Source	Country (income level)^a^	Level of hospital^b^	Scale of pathway	Surgical urgency^b^	Surgical specialty^c^	Comparator^d^	Number of patients
*Quantitative descriptive studies*							
Agarwal et al. 2018 [25]	India (LM)	3rd	Hospital	Elective	HPB	None	394
Ahmed et al. 2010 [26]	Pakistan (LM)	3rd	Hospital	Mixed	CT	None	274
Akhtar et al. 2000 [27]	Pakistan (LM)	3rd	Single team	Elective	CT	None	150
Chaudhary et al. 2015 [28]	India (LM)	3rd	Hospital	Elective	HPB	None	208
Jain et al. 2015 [29]	India (LM)	3rd	Hospital	Emergency	T&O	None	119
Kulshrestha et al. 2019 [30]	India (LM)	NR	Hospital	Emergency	T&O	None	114
Mahendran et al. 2019 [31]	India (LM)	3rd	Single team	Elective	HPB	None	50
Mangukia et al. 2019 [32]	India (LM)	3rd	Hospital	Mixed	CT	None	709
Pandit et al. 2019 [33]	Nepal (L)	3rd	Single team	Elective	HPB	None	25
Vashistha et al. 2018 [34]	India (LM)	3rd	Hospital	Emergency	CR; UGI	None	102
*Quantitative non-randomized studies*							
Khowaja 2006 [35]	Pakistan (LM)	3rd	Hospital	Elective	Uro	Previous SOC	200
Kurmi et al. 2020 [36]	Nepal (L)	3rd	Single team	Elective	CR	SOC in another surgical ward	30
Kuzmenko et al. 2019 [37]	Ukraine (LM)	3rd	Single team	NR	HPB	Previous SOC	78
Nanavati and Prabhakar 2014 [38]	India (LM)	3rd	Hospital	Elective	CR	Previous SOC	60
Nanavati and Prabhakar 2015 [39]	India (LM)	3rd	Hospital	Elective	CR	Previous SOC	50
Pal et al. 2003 [40]	Pakistan (LM)	3rd	Hospital	Elective	HPB	Previous SOC	106
Pillai et al. 2014 [41]	India (LM)	3rd	Hospital	Elective	HPB	Previous SOC	40
Quader et al. 2010 [42]	Bangladesh (LM)	3rd	Single team	Elective	CT	SOC in another surgical ward	50
Sahoo et al. 2014 [43]	India (LM)	3rd	Hospital	Elective	UGI	Previous SOC	47
Sanad et al. 2019 [44]	Egypt, Arab Rep. (LM)	3rd	Hospital	NR	O&G	Previous SOC	58
Shah et al. 2016 [45]	India (LM)	3rd	Single team	Elective	HPB	Previous SOC	188
Shrikhande et al. 2013 [46]	India (LM)	3rd	Hospital	Elective	HPB	Previous SOC; Earlier version of pathway	500
*Quantitative randomized controlled trials*							
Baluku et al. 2020 [47]	Uganda (L)	3rd	Hospital	Emergency	O&G	Previous SOC	160
Bansal et al. 2020 [48]	India (LM)	3rd	Hospital	Elective	Uro	Previous SOC	54
Iyer and Kareem 2019 [49]	India (LM)	2nd	Hospital	Elective	CR	Previous SOC	100
Pirzada et al. 2017 [50]	Pakistan (LM)	NR	Hospital	Elective	CR	Previous SOC	60
Shetiwy et al. 2017 [51]	Egypt, Arab Rep. (LM)	3rd	Hospital	Elective	CR	Previous SOC	70

^a^*L* Low, *LM* Lower-middle

^b^*NR* Not reported

^c^*Br*, Breast, *CR* Colorectal, *CT* Cardiothoracic, *HPB* Hepato-pancreaticobiliary, *O&G* Obstetrics & Gynecology, *T&O* Trauma & Orthopedics, *UGI* Upper Gastrointestinal, *Uro* Urology

^d^*SOC* Standard of care

The majority of studies were set in third-level institutions (*n* = 24, 89%), while none were from first-level institutions. Twenty-one articles (78%) reported pathways implemented at a hospital-wide scale. The other six (22%) were at a single perioperative team scale. The specialties in which care pathways were most commonly studied were hepato-pancreaticobiliary (*n* = 9, 33%), colorectal (*n* = 7, 26%) and cardiothoracic (*n* = 4, 15%). The majority of articles reported care pathways for elective surgery (*n* = 20, 74%). One (4%) article reported exclusively on a pediatric (≤ 18 years) pathway.

The design of included studies was quantitative non-randomized for 12 (44%), quantitative descriptive for 10 (37%) and quantitative randomized controlled for 5 (19%). There were no qualitative or mixed-method studies. Seventeen (63%) studies evaluated pathways against a comparator, most commonly (*n* = 14) previous standard of care.

### Critical appraisal within sources of evidence

Most studies (*n* = 19, 70%) were of low (MMAT score = 0–2) or medium quality (MMAT score = 3) as outlined in Table 2. Common limitations were failure to meet the criteria ‘Did the participants adhere to the assigned intervention’ and ‘During the study period, is the intervention administered as intended’ for randomized controlled and non-randomized studies, respectively. None of the 5 randomized controlled trials demonstrated that outcome assessors were blinded to the intervention.

**Table 2:** Mixed Methods Appraisal Tool (MMAT) quality ratings for each study

Source	MMAT Criteria (0, Can't tell or no; 1, Yes)^a^															Overall score
	1.1	1.2	1.3	1.4	1.5	2.1	2.2	2.3	2.4	2.5	3.1	3.2	3.3	3.4	3.5	
Agarwal et al. 2018 [25]											1	1	1	1	1	*****
Ahmed et al. 2010 [26]											1	0	0	1	1	***
Akhtar et al. 2000 [27]											1	0	0	0	0	*
Baluku et al. 2020 [47]	1	0	1	0	0											**
Bansal et al. 2020 [48]	1	1	1	0	0											***
Chaudhary et al. 2015 [28]											1	1	0	1	1	****
Iyer and Kareem 2019 [49]	0	0	0	0	0											0
Jain et al. 2015 [29]											1	0	1	0	1	***
Khowaja 2006 [35]						0	0	1	0	0						*
Kulshrestha et al. 2019 [30]											1	1	1	1	1	*****
Kurmi et al. 2020 [36]						1	1	1	1	0						****
Kuzmenko et al. 2019 [37]						0	1	1	1	0						***
Mahendran et al. 2019 [31]											1	1	0	0	0	**
Mangukia et al. 2019 [32]											1	1	1	0	1	****
Nanavati and Prabhakar 2014 [38]						1	1	1	0	0						***
Nanavati and Prabhakar 2015 [39]						1	1	0	0	0						**
Pal et al. 2003 [40]						0	1	1	0	1						***
Pandit et al. 2019 [33]											1	0	1	0	1	***
Pillai et al. 2014 [41]						0	0	1	0	0						*
Pirzada et al. 2017 [50]	1	0	1	0	0											**
Quader et al. 2010 [42]						0	1	1	1	0						***
Sahoo et al. 2014 [43]						1	1	1	1	0						****
Sanad et al. 2019 [44]						0	1	0	0	1						**
Shah et al. 2016 [45]						1	1	1	1	1						*****
Shetiwy et al. 2017 [51]	0	1	1	0	0											**
Shrikhande et al. 2013 [46]						1	1	0	1	0						***
Vashistha et al. 2018 [34]											1	1	1	0	1	****

^a^1. For quantitative randomized controlled trials

1.1. Is randomization appropriately performed?

1.2. Are the groups comparable at baseline?

1.3. Are there complete outcome data?

1.4. Are outcome assessors blinded to the intervention provided?

1.5 Did the participants adhere to the assigned intervention?

2. For quantitative non-randomized

2.1. Are the participants representative of the target population?

2.2. Are measurements appropriate regarding both the outcome and intervention (or exposure)?

2.3. Are there complete outcome data?

2.4. Are the confounders accounted for in the design and analysis?

2.5. During the study period, is the intervention administered (or exposure occurred) as intended?

3. For quantitative descriptive

3.1. Is the sampling strategy relevant to address the research question?

3.2. Is the sample representative of the target population?

3.3. Are the measurements appropriate?

3.4. Is the risk of nonresponse bias low?

3.5. Is the statistical analysis appropriate to answer the research question?

### Pathway design and clinical interventions

Twenty-three (85%) of the included articles reported ‘adapted’ pathways. Almost all of these referenced ERAS (Enhanced Recovery After Surgery) or Fast-track guidelines as the original source. Two (7%) described pathways that were designed de novo. While fulfilling the inclusion criteria, one study did not provide details of pathway interventions [35]. Owing to the heterogeneity of pathways, no attempt was made to synthesize the nature of reported clinical interventions; however, these are listed in Online Resource 4.

### Study aims and outcomes

Five articles (19%) referred to the evaluation of ‘safety’ within the title or study aim and three (11%) used the term ‘feasibility’. Table 3 summarizes the reported outcomes. A total of 375 outcome measures were charted across 27 articles. Of these, physiological and clinical outcomes were most common (*n* = 182, 49%). Twelve studies (44%) reported a physical functioning outcome, of which most related to early postoperative milestones of drinking, eating and mobilizing. Besides pain assessment, there were only three (1%) patient-reported outcome measures (PROMS); two studies reported mobility scores and one assessed patient satisfaction [29, 30, 35].

**Table 3:** Summary of outcomes categorized according to the COMET taxonomy [23]

Core area	Outcome domain	Overall frequency, *n*	No. of articles reporting at least one outcome within domain, *n* (%)
Death	Death—Mortality/survival	20	19 (70.4%)
Physiological/clinical^a^	Physiological/clinical	182	26 (96.3%)
Life impact	Physical functioning	17	12 (44.4%)
	Social functioning	0	0 (0%)
	Role functioning	0	0 (0%)
	Emotional functioning/wellbeing	0	0 (0%)
	Cognitive functioning	0	0 (0%)
	Global quality of life	0	0 (0%)
	Perceived health status	0	0 (0%)
	Delivery of care	26	10 (37%)
	Personal circumstance	0	0 (0%)
Resource use	Economic	4	4 (14.8%)
	Hospital	51	26 (96.3%)
	Need for further intervention	61	22 (81.5%)
	Societal/carer burden	0	0 (0%)
Adverse events	Adverse events/effects	14	14 (51.9%)

^a^Physiological/clinical outcome domains have been grouped owing to the heterogeneity of surgical specialties

Most studies reported a hospital resource use outcome measure (*n* = 26, 96%), with 25 studies reporting length of hospital stay and 18 reporting readmission rates. Eight articles (30%) described adherence to intervention as an outcome measure. Some provided an overall statistic for compliance; however, only one study offered a detailed breakdown of the adherence to all pathway components [25].

### Pathway implementation strategies

The number of implementation strategies reported by each study ranged from 0 to 9 (median = 2). No strategies were reported in 4 articles (15%). The most frequently reported strategy within each ERIC taxonomy cluster is shown in Table 4. Across 27 articles, 24 of the 73 ERIC strategies were used. The most frequently reported strategies were “Prepare patients/consumers to be active participants” and “Promote adaptability”. There were no strategies that targeted an infrastructure change.

**Table 4:** Summary of implementation strategies categorized according to the Expert Recommendations for Implementing Change (ERIC) classification [21, 22]

Strategy cluster	No. of articles reporting at least one strategy within cluster, *n* (%)	Most frequently reported strategy within the cluster, n
Use evaluative and iterative strategies	9 (33%)	Assess for readiness and identify barriers and facilitators, *n* = 3 Purposefully re-examine the implementation, *n* = 3 Stage implementation scale up, *n* = 3
Provide interactive assistance	2 (7%)	Facilitation, *n* = 2
Adapt and tailor to context	11 (41%)	Promote adaptability, *n* = 11
Develop stakeholder interrelationships	6 (22%)	Build a coalition, *n* = 4
Train and educate stakeholders	2 (7%)	Distribute educational materials, *n* = 2
Support clinicians	6 (22%)	Create new clinical teams, *n* = 3
Engage consumers	19 (70%)	Prepare patients/consumers to be active participants, *n *= 18
Utilize financial strategies	1 (4%)	Access new funding, *n* = 1
Change infrastructure	0 (0%)	N/A

### Facilitators and barriers to pathway implementation

Implementation facilitators and barriers according to CFIR construct are summarized in Table 5.

**Table 5:** Summary of implementation barriers and facilitators categorized according to the Consolidated Framework for Implementation Research (CFIR) [24]

CFIR domain	CFIR constructs	
	Facilitators of implementation	Barriers to implementation
Intervention characteristics	Evidence strength and quality Adaptability Trialability	Cost
Outer setting	External policy and incentives Peer pressure Patient needs and resources	Patient needs and resources
Inner setting	Networks and communications Tension for change	Available resources
Characteristics of individuals	–	Knowledge and beliefs about the intervention
Process	Planning Formally appointed internal implementation leaders	–

#### Intervention characteristics

Most articles framed existing literature, almost exclusively from HICs, as a facilitator for implementation and adapted published pathways to the setting and type of surgery. For example, a pathway for pancreatic cancer resections adapted from ERAS recommendations omitted selective preoperative biliary drainage as this was performed elsewhere prior to admission [25]. The ability to trial a pathway on a smaller scale served as a facilitator as some expanded the use of pathways to other types of surgery after first implementing and evaluating a single pathway [27]. Others evaluated a new pathway against current care so that the better model could be used [50]. While a reduction in cost and resource use was a commonly cited advantage of pathway implementation, the cost of interventions was often a barrier. One study reported that financial constraints in Punjab province meant that minimally invasive surgery could not be offered [27]. In India, carbohydrate drinks recommended by ERAS were not commercially available [48], while thromboprophylaxis and ondansetron (antiemetic) could not be offered in Uganda as these were too expensive [47].

#### Outer setting

International guidelines, particularly by the ERAS society, were incorporated into most reviewed pathways and were an important facilitator. One study reported pressure to conform to international standards as a driver for implementation [26]. Institutional prioritization of patient needs facilitated the implementation of some pathways. Earlier return to work afforded by the pathway was cited as a priority for patients and thus an important reason for implementing fast track surgery in an Indian study [39]. A study from Pakistan recognized that when a child is admitted for surgery, the whole family moves close to the hospital, incurring a cost for accommodation [27]. Minimizing the length of hospital stay was therefore hoped to reduce costs for the family. Conversely, a study from Bangladesh described that lack of follow-up services outside the city led patients in the ‘fast track’ pathway to stay near the hospital for at least a week post-discharge [42].

#### Inner setting

Cooperation and good team communication were reported as facilitators to implementation [25, 27]. High demand for surgery, performance indicators and the need for efficient use of limited resources created tension for change away from existing care [25, 42, 50]. Two Indian studies conducted in specialized hospitals acknowledged that their existing resources were not representative of most LMIC institutions, where resource constraints could act as a barrier [30, 34]. Indeed, a lack of human resources and funding coupled with increasing patient numbers acted as a barrier in another Indian study [25].

#### Characteristics of individuals

Several studies hypothesized that clinician beliefs were the reason pathways were not widely implemented within their countries [33, 38, 48], though this was not reported as a barrier in their institutions. However, early discharge and outpatient surgery were hindered by surgeons’ conservative approach as well as patients’ fear of leaving the safety of a hospital [31, 42].

#### Process

Literature searches and multidisciplinary consensus meetings were used to design care pathways [29, 40]. Implementation was facilitated by the establishment of improvement teams and allocation of formal roles, including a dedicated supervisor for the entire pathway [30, 44, 46]. Senior clinicians with influence within a department often led implementation [28, 34, 45]. One study reported the use of a formal implementation methodology, the King’s interacting systems framework and theory of goal attainment, as a facilitator [35]. Although all studies reported quantitative outcomes, only one described how this information was used to aid further improvement [28].

## Discussion

In this systematic review, we identified 27 studies published between 2000 and 2020 addressing the implementation and evaluation of perioperative care pathways in low and lower-middle-income countries. The main review finding is the sparsity of literature from low-income countries and first-level hospitals focused on emergency surgery. Existing studies reveal increasing evaluation of perioperative pathways, adapted to the realities of LMICs, to improve quality and reduce costs in a geographically diverse set of countries. Additionally, this review found a limited number of high-quality studies, lack of detail regarding adherence to pathway components, and absence of concurrent qualitative data collection to facilitate a deeper understanding of pathway implementation.

Ensuring access to essential surgical care is a key target outlined by the Lancet Commission and is measured as access to a facility that is able to perform cesarean delivery, laparotomy and open fracture repair (Bellwether Procedures) [1, 52]. Therefore, it is concerning that our review identified only two articles studying the implementation of a Bellwether Procedure pathway in settings with the highest need for essential surgical care. Furthermore, most pathways included in this review aimed to standardize care for complex elective procedures in third-level hospitals. This limits learning that such studies may offer others in similar resource-constrained contexts, such as those working to address the three times higher risk-adjusted mortality from emergency abdominal surgery or 50 times higher maternal mortality rates following cesarean section in LMICs compared to HICs [2, 53].

This poor perioperative care quality in LMICs is conceptualized to occur due to failures to provide timely access to services, deliver safe care and rescue post-operatively [13], and thus understanding how care pathways might overcome these failures in low-resource settings would be beneficial. Unfortunately, in our review, there was poor reporting by most authors about the process, facilitators and barriers to implementation. Despite this, we found similar themes with authors of a systematic review from HICs, who identified adapting pathways to fit the local context and resistance from frontline clinicians as major facilitators and barriers, respectively [54].

Differences in adherence to pathway components could highlight further context-specific facilitators and barriers. Unfortunately, only one study in this review reported the rate of adherence to all interventions within the pathway [25]. Poor compliance reporting is common and not confined to LMICs [55]. Thus, the ERAS society now recommends a standardized framework for reporting compliance [56], which were the most common source for pathways in the included articles. Adopting this framework would improve reporting quality, which was generally low or medium in this review.

Included articles mostly did not report on patient-reported outcome measures. Patient experience is increasingly recognized as one of the three pillars of quality alongside effectiveness and safety, and initiatives focused on enhancing patient experience have shown to lead to better levels of quality [57, 58]. Understanding patient experiences of perioperative pathways in LMICs would help ensure pathways are fit for purpose.

This study has strengths and limitations. Exclusion of non-English articles, grey literature and studies from UMICs may have excluded articles set in resource-poor institutions relevant to this review. Articles may have also been missed due to the varied nomenclature used for care pathways. However, these limitations were mitigated by our comprehensive search strategy, use of multiple databases, acquisition of 3 further articles from contacting authors directly and taking an inclusive approach during screening; factors which we feel are a strength of this review. Articles from UMICs will be reviewed separately to offer further learning to relevant contexts (CRD42022324301). Lastly, half of the sample was made up of articles from India. Despite being from the same country, the studies were conducted in diverse institutions, including public and private and second-level and third-level hospitals, and offered different and valuable insights into pathway implementation.

## Conclusions

This systematic review presents an overview of literature on perioperative care pathways in low and lower-middle-income countries and offers a starting point for further applied health services research. Perioperative pathway implementation in LMICs has been increasingly reported in the literature with details regarding adaptations needed to ensure they are feasible in resource-limited settings. Future work may consider studying pathways for procedures with broader relevance within LMICs (e.g., Bellwether Procedures) and using standardized frameworks to improve reporting quality. Furthermore, qualitative and implementation research, including on adherence and patient experiences, would make a valuable contribution to existing knowledge and help improve patient outcomes.

## Supplementary Information

Below is the link to the electronic supplementary material.

Supplementary file1 (PDF 106 KB)

Supplementary file2 (PDF 84 KB)

Supplementary file3 (PDF 77 KB)

Supplementary file4 (PDF 314 KB)

## Data Availability

The data extracted from the included studies and data used for all analyses are available in Online Resource 4. The template data collection form can be obtained from the authors on request.

## Declarations

### Systematic review registration

PROSPERO CRD42020172978.
