# Supplementary material for: Perioperative Care Pathways in Low- and Lower-Middle-Income Countries: Systematic Review and Narrative Synthesis
Source: World J Surg. 2022 Jun 22;46(9):2102–13. doi: 10.1007/s00268-022-06621-x (PMC9334384; doi:10.1007/s00268-022-06621-x)
Supplement: Supplementary file 2 — Supplementary file2 (PDF 84 KB) [file 268_2022_6621_MOESM2_ESM.pdf]

Perioperative Care Pathways in Low- and Lower-Middle-Income Countries: Systematic Review and Narrative Synthesis

Authors: Jignesh Patel, Timo Tolppa, Bruce M. Biccard, Brigitta Fazzini, Rashan Haniffa, Debora Marletta, Ramani Moonesinghe, Rupert Pearse, Sutharshan Vengadasalam, Timothy J. Stephens and Cecilia Vindrola-Padros

Journal name: World Journal of Surgery

Corresponding author: Timothy J. Stephens; Critical Care and Perioperative Medicine Research Group, Queen Mary University of London, London, UK; t.t.stephens@qmul.ac.uk

Online Resource 2 Search terms for MEDLINE database

Search constructs: [LMIC] AND [Perioperative] AND [Care pathway]

Search terms:

[LMIC]

Developing Countries/ or ((developing or less* developed or under developed or underdeveloped or middle income or low* income or low* resource or deprived or poor*) adj (countr* or nation?)).ti,ab,kw. or (low adj3 middle adj3 countr*).ti,ab,kw. or LMIC*.ti,ab,kw. or LAMI*.ti,ab,kw. or third world.ti,ab,kw. or transitional countr*.ti,ab,kw. or global south.ti,ab,kw. or Africa/ or Africa.ti,ab,kw. or Central America.ti,ab,kw. or Central America/ or afghanistan.ti,ab,kw. or Afghanistan/ or Albania/ or albania.ti,ab,kw. or algeria.ti,ab,kw. or Algeria/ or American Samoa.ti,ab,kw. or American Samoa/ or angola.ti,ab,kw. or Angola/ or argentina.ti,ab,kw. or Argentina/ or armenia.ti,ab,kw. or Armenia/ or Azerbaijan.ti,ab,kw. or Azerbaijan/ or bangladesh.ti,ab,kw. or Bangladesh/ or belarus.ti,ab,kw. or "Republic of Belarus"/ or Belorussia.ti,ab,kw. or belize.ti,ab,kw. or Belize/ or benin.ti,ab,kw. or Benin/ or dahomey.ti,ab,kw. or Bhutan/ or bhutan.ti,ab,kw. or bolivia.ti,ab,kw. or Bolivia/ or bosnia.ti,ab,kw. or "Bosnia and Herzegovina"/ or Herzegovina.ti,ab,kw. or botswana.ti,ab,kw. or Botswana/ or Bechuanaland.ti,ab,kw. or Kalahari.ti,ab,kw. or brazil.ti,ab,kw. or Brazil/ or Bulgaria.ti,ab,kw. or Bulgaria/ or

burkina faso.ti,ab,kw. or Burkina Faso/ or Burkina Fasso.ti,ab,kw. or Upper Volta.ti,ab,kw. or Burundi.ti,ab,kw. or Burundi/ or Cape Verde.ti,ab,kw. or Cabo Verde/ or cabo verde.ti,ab,kw. or cambodia.ti,ab,kw. or Cambodia/ or Cameroon.ti,ab,kw. or Cameroon/ or Central African Republic/ or Central African republic.ti,ab,kw. or ubangi-shari.ti,ab,kw. or ubangi shari.ti,ab,kw. or chad.ti,ab,kw. or Chad/ or china.ti,ab,kw. or China/ or Colombia.ti,ab,kw. or Colombia/ or Comoros.ti,ab,kw. or Comoros/ or comoro islands.ti,ab,kw. or Mayotte.ti,ab,kw. or Iles Comores.ti,ab,kw. or Democratic republic of the Congo.ti,ab,kw. or "Democratic Republic of the Congo"/ or (democratic republic adj2 Congo).ti,ab,kw. or Belgian Congo.ti,ab,kw. or Zaire.ti,ab,kw. or congo.ti,ab,kw. or Congo/ or Congo Red/ or congo red.ti,ab,kw. or crimean-congo.ti,ab,kw. or Costa rica.ti,ab,kw. or Costa Rica/ or cote d'ivoire.ti,ab,kw. or Cote d'Ivoire/ or Ivory Coast.ti,ab,kw. or Cuba.ti,ab,kw. or Cuba/ or Djibouti/ or Djibouti.ti,ab,kw. or French Somaliland.ti,ab,kw. or Dominica.ti,ab,kw. or Dominica/ or Dominican republic.ti,ab,kw. or Dominican Republic/ or ecuador.ti,ab,kw. or Ecuador/ or Egypt/ or Egypt.ti,ab,kw. or El Salvador/ or el salvador.ti,ab,kw. or Equatorial Guinea.ti,ab,kw. or Equatorial Guinea/ or spanish guinea.ti,ab,kw. or Eritrea.ti,ab,kw. or Eritrea/ or Swaziland/ or eswatini.ti,ab,kw. or swaziland.ti,ab,kw. or ethiopia.ti,ab,kw. or Ethiopia/ or Fiji.ti,ab,kw. or Fiji/ or gabon.ti,ab,kw. or Gabon/ or gabonese republic.ti,ab,kw. or gambia.ti,ab,kw. or Gambia/ or "Georgia (Republic)"/ or Georgia/ or Georgia.ti,ab,kw. or Ghana.ti,ab,kw. or Ghana/ or Gold Coast.ti,ab,kw. or grenada.ti,ab,kw. or Grenada/ or guatemala.ti,ab,kw. or Guatemala/ or (guinea not (New Guinea or guinea pig* or guinea fowl)).ti,ab,kw. or Guinea/ or guinea-bissau.ti,ab,kw. or Guinea-Bissau/ or Portuguese guinea.ti,ab,kw. or guyana.ti,ab,kw. or Guyana/ or haiti.ti,ab,kw. or Haiti/ or honduras.ti,ab,kw. or Honduras/ or India.ti,ab,kw. or India/ or Indonesia.ti,ab,kw. or Indonesia/ or iran.ti,ab,kw. or Iran/ or Iraq/ or iraq.ti,ab,kw. or jamaica.ti,ab,kw. or Jamaica/ or Jordan.ti,ab,kw. or Jordan/ or Kazakhstan.ti,ab,kw. or Kazakhstan/ or Kenya.ti,ab,kw. or Kenya/ or kiribati.ti,ab,kw. or Micronesia/ or "Democratic People's Republic of Korea"/ or (north korea or (democratic people* republic adj2 Korea)).ti,ab,kw. or kosovo.ti,ab,kw. or Kosovo/ or kyrgyz Republic.ti,ab,kw. or Kirghizia.ti,ab,kw. or Kirghiz.ti,ab,kw. or Kyrgyzstan.ti,ab,kw. or Kyrgyzstan/ or laos.ti,ab,kw. or Laos/ or (lao adj1 democratic

republic) .ti,ab,kw. or lebanon.ti,ab,kw. or Lebanon/ or Lesotho.ti,ab,kw. or Lesotho/ or basutoland.ti,ab,kw. or Liberia.ti,ab,kw. or Liberia/ or Libya/ or libya.ti,ab,kw. or madagascar.ti,ab,kw. or Madagascar/ or Malagasy republic.ti,ab,kw. or malawi.ti,ab,kw. or Malawi/ or nyasaland.ti,ab,kw. or malaysia.ti,ab,kw. or Malaysia/ or maldives.ti,ab,kw. or mali.ti,ab,kw. or Mali/ or Marshall island*.ti,ab,kw. or Caroline island*.ti,ab,kw. or Ellie island*.ti,ab,kw. or Gilbert island*.ti,ab,kw. or Johnston island*.ti,ab,kw. or Mariana island*.ti,ab,kw. or pacific island*.ti,ab,kw. or mauritania.ti,ab,kw. or Mauritania/ or Mauritius.ti,ab,kw. or Mauritius/ or agalega islands.ti,ab,kw. or Mexico/ or mexico.ti,ab,kw. or moldova.ti,ab,kw. or Moldova/ or mongolia.ti,ab,kw. or Mongolia/ or montenegro.ti,ab,kw. or Montenegro/ or morocco.ti,ab,kw. or Morocco/ or mozambique.ti,ab,kw. or Mozambique/ or Mocambique.ti,ab,kw. or Portuguese east africa.ti,ab,kw. or myanmar.ti,ab,kw. or Myanmar/ or burma.ti,ab,kw. or namibia.ti,ab,kw. or Namibia/ or nauru.ti,ab,kw. or nepal.ti,ab,kw. or Nepal/ or nicaragua.ti,ab,kw. or Nicaragua/ or Niger/ or (niger not (Aspergillus or Peptococcus or Schizothorax or Cruciferae or Gobius or Lasius or Agelastes or Melanosuchus or radish or Parastromateus or Orius or Apergillus or Parastromateus or Stomoxys)).ti,ab,kw. or nigeria.ti,ab,kw. or Nigeria/ or north macedonia.ti,ab,kw. or "Macedonia (Republic)"/ or macedonia.ti,ab,kw. or pakistan.ti,ab,kw. or Pakistan/ or papua new guinea.ti,ab,kw. or Papua New Guinea/ or paraguay.ti,ab,kw. or Paraguay/ or peru.ti,ab,kw. or Peru/ or philippines.ti,ab,kw. or Philippines/ or romania.ti,ab,kw. or Romania/ or exp Russia/ or Russia.ti,ab,kw. or ussr.ti,ab,kw. or Russian federation.ti,ab,kw. or union of soviet socialist republics or Soviet Union.ti,ab,kw. or rwanda.ti,ab,kw. or Rwanda/ or ruanda.ti,ab,kw. or "Independent State of Samoa"/ or samoa.ti,ab,kw. or Samoa/ or navigator island*.ti,ab,kw. or samoan island*.ti,ab,kw. or sao tome.ti,ab,kw. or (sao tome adj2 principe).ti,ab,kw. or senegal.ti,ab,kw. or Senegal/ or serbia.ti,ab,kw. or Serbia/ or Sierra leone.ti,ab,kw. or Sierra Leone/ or Solomon island*.ti,ab,kw. or somalia.ti,ab,kw. or Somalia/ or south africa.ti,ab,kw. or South Africa/ or south sudan.ti,ab,kw. or South Sudan/ or Sri Lanka/ or sri lanka.ti,ab,kw. or st lucia.ti,ab,kw. or Saint Lucia/ or saint lucia.ti,ab,kw. or Saint Vincent.ti,ab,kw. or grenadines.ti,ab,kw. or "Saint Vincent and the Grenadines"/ or Sudan/ or sudan.ti,ab,kw. or suriname.ti,ab,kw. or Suriname/ or syria.ti,ab,kw. or Syria/ or syrian arab

republic.ti,ab,kw. or Tajikistan/ or tajikistan.ti,ab,kw. or Tanzania.ti,ab,kw. or Tanzania/ or tanganyika.ti,ab,kw. or zanzibar.ti,ab,kw. or thailand.ti,ab,kw. or Thailand/ or timor-leste.ti,ab,kw. or Timor-Leste/ or togo.ti,ab,kw. or Togo/ or togolese republic.ti,ab,kw. or tongo.ti,ab,kw. or tunisia.ti,ab,kw. or Tunisia/ or Turkey/ or (turkey.ti,ab,kw. not animal/) or turkmenistan.ti,ab,kw. or Turkmenistan/ or tuvalu.ti,ab,kw. or uganda.ti,ab,kw. or Uganda/ or ukraine.ti,ab,kw. or Ukraine/ or uzbekistan.ti,ab,kw. or Uzbekistan/ or vanuatu.ti,ab,kw. or Vanuatu/ or venezuela.ti,ab,kw. or Venezuela/ or vietnam.ti,ab,kw. or Vietnam/ or viet nam.ti,ab,kw. or west bank.ti,ab,kw. or gaza.ti,ab,kw. or yemen.ti,ab,kw. or Yemen/ or zambia.ti,ab,kw. or Zambia/ or northern Rhodesia.ti,ab,kw. or zimbabwe.ti,ab,kw. or Zimbabwe/ or rhodesia.ti,ab,kw.

[Perioperative]
exp Perioperative Care/ or Perioperative Medicine/ or exp Perioperative Period/ or perioperative.ti,ab,kw. or peri-operative.ti,ab,kw. or peroperative.ti,ab,kw. or per-operative.ti,ab,kw. or Preoperative Care/ or preoperative.ti,ab,kw. or pre-operative.ti,ab,kw. or exp Specialties, Surgical/ or surgery.ti,ab,kw. or surgical.ti,ab,kw. or operative.ti,ab,kw. or exp Surgical Procedures, Operative/ or postoperative.ti,ab,kw. or post-operative.ti,ab,kw.

[Care pathway]
Critical Pathways/ or Clinical Protocols/ or pathway?.ti,ab,kw. or ((care or clinical or critical or practice or treatment) adj (path? or map? or protocol?)).ti,ab,kw. or ((care or clinical or practice or treatment) adj (algorithm)).ti,ab,kw. or fast track.ti,ab,kw. or accelerated recovery.ti,ab,kw. or ERAS.ti,ab,kw. or enhanced recovery.ti,ab,kw.
