# Supplementary material for: Perioperative Care Pathways in Low- and Lower-Middle-Income Countries: Systematic Review and Narrative Synthesis
Source: World J Surg. 2022 Jun 22;46(9):2102–13. doi: 10.1007/s00268-022-06621-x (PMC9334384; doi:10.1007/s00268-022-06621-x)
Supplement: Supplementary file 3 — Supplementary file3 (PDF 77 KB) [file 268_2022_6621_MOESM3_ESM.pdf]

Perioperative Care Pathways in Low- and Lower-Middle-Income Countries: Systematic Review and Narrative Synthesis

Authors: Jignesh Patel, Timo Tolppa, Bruce M. Biccard, Brigitta Fazzini, Rashan Haniffa, Debora Marletta, Ramani Moonesinghe, Rupert Pearse, Sutharshan Vengadasalam, Timothy J. Stephens and Cecilia Vindrola-Padros

Journal name: World Journal of Surgery

Corresponding author: Timothy J. Stephens; Critical Care and Perioperative Medicine Research Group, Queen Mary University of London, London, UK; t.t.stephens@qmul.ac.uk

Online Resource 3 Definition of the levels of hospital care

Taken from McCord C, Ozgediz D, Beard JH, Debas HT (2016) General Surgical Emergencies. In: Debas HT, Donkor P, Gawande A, et al (eds) Essential Surgery: Disease Control Priorities, Third Edition (Volume 1). The International Bank for Reconstruction and Development / The World Bank, Washington (DC)

Level of hospital care	Definition
First-level hospital	Few specialties; mainly internal medicine, obstetrics and gynecology, pediatrics, and general surgery.<br>Often only one general practice physician or a nonphysician clinician.<br>Limited laboratory services available for general analysis but not for specialized pathological analysis<br>50–250 beds
Second-level hospital	More differentiated by function, with as many as 5 to 10 clinical specialties.<br>200–800 beds
Third-level hospital	Highly specialized staff and technical equipment (e.g. cardiology, intensive care unit, and specialized imaging units)<br>Clinical services highly differentiated by function<br>Teaching activities in some facilities

300–1,500 beds
