# Supplementary material for: Perioperative Care Pathways in Low- and Lower-Middle-Income Countries: Systematic Review and Narrative Synthesis
Source: World J Surg. 2022 Jun 22;46(9):2102–13. doi: 10.1007/s00268-022-06621-x (PMC9334384; doi:10.1007/s00268-022-06621-x)
Supplement: Supplementary file 4 — Supplementary file4 (PDF 314 KB) [file 268_2022_6621_MOESM4_ESM.pdf]

Perioperative Care Pathways in Low- and Middle-Income Countries: Systematic Review and Narrative Synthesis

Authors: Jignesh Patel, Timo Tolppa, Bruce M. Biccard, Rashan Haniffa, Debora Marletta, Ramani Moonesinghe, Rupert Pearse, Sutharshan Vengadasalam, Timothy J. Stephens and Cecilia Vindrola-Padros

Journal name: World Journal of Surgery

Corresponding author: Timothy J. Stephens; Critical Care and Perioperative Medicine Research Group, Queen Mary University of London, London, UK;

t.t.stephens@qmul.ac.uk

Online Resource 4 Raw Data

Table 1 The design and clinical components of care pathways (where appropriate, direct quotations have been used)

Source	Pathway Design	Pre-operative interventions	Intra-operative interventions	Post-operative interventions
Agarwal et al, 2018 [1]	adapted	1. Counselling<br>2. Selective nutritional supplements and high-protein diet<br>3. Physiotherapy and exercise training<br>4. Venous thromboembolism prophylaxis<br>5. Carbohydrate drink night before surgery<br>6. Carbohydrate drink 2 h before surgery<br>7. Selective bowel preparation<br>8. Selective sedative premedication	1. Mid-thoracic epidural<br>2. Antibiotic prophylaxis 1 h before incision<br>3. Maintenance of normothermia<br>4. Antiemetic prophylaxis	1. Epidural analgesia or patient-controlled analgesia<br>2. Multimodal analgesia with paracetamol and NSAIDs<br>3. Mobilisation started on POD1<br>4. Liquids initiated on POD1<br>5. Mashed diet by POD5
Ahmed et al, 2010 [2]	adapted	1. Preoperative education<br>2. Premedication (lorazepam 2-3mg PO)	1. standard surgical practice: minimise cross-clamp &bypass time. Cold blood cardioplegia was used for valvular, adult congenital cases, combined coronary&valvular procedures.CABG was mainly done with intermittent cross clamp-fibrillation technique.<br>2. mild hypothermia<br>3. Autologous blood transfusions<br>4. Standardised anaesthetic protocol: Co-induction with morphine 0.3–0.5 mg/Kg, midazolam 0.04–0.06 mg/Kg and vecuronium 0.02–0.04 mg/Kg. Amnesia induced using propofol boluses 5–10 ml. Anaesthesia was maintained with oxygen, air, sevoflourane 1–2% and vecuronium 1–2 mg. Active monitoring according to the PIMS cardiac anaesthesia protocol. Peri- operatively low to moderate doses of opioids were used instead of high doses. Thoracic epidural anaesthesia according to standard practices.	1. Muscle paralysing or sedative agents were avoided whenever possible<br>2. All patients were shifted to cardiac ICU and monitored haemodynamically<br>3. All the biochemical investigations and blood gases were repeated, and temperatures maintained above 36 degrees central.<br>4. Blood loss was monitored to remain below 150–100 ml/hour.<br>5. Extubation criteria was awake patient with adequate muscle strength, Fio2 less than 50%, Tidal volume more than 5 ml/Kg, Vital capacity more than 10 ml/Kg, respiratory rate less than 30 and satisfactory arterial blood gases.<br>6. Active respiratory and physical training from postoperative day one.

Akhtar et al, 2000 [3]	adapted	1. preoperative outpatient review by surgeon, cardiac anaesthetist and intensive care sister.<br>2. Routine blood investigations, chest x-ray, blood grouping and cross matching<br>3. The procedure was explained to the parents or the patient in detail<br>4. Nil by mouth instructions (NPO) were given to the parents. Patients were allowed a light snack at midnight following which they were to be kept NPO, infants and small children were allowed a last mothers feed at 2:00am<br>5. patients asked to report to the intensive care unit (ICU) at 0700hrs where their vital signs were taken and necessary consent forms were signed. If patients were unable to reach by 7:00am adjustments were made to suit the patient and the operation was scheduled for the afternoon).<br>6. Premedication of promethazine hydrochloride 15-20mg.	1. Standard anaesthetic technique (thiopentone 3mg/kg, Tracrium 0.6mg/kg)<br>2. ECG, pulse oximetry and invasive blood pressure monitoring (in patients over 18years). In the younger age group blood pressure was monitored by a non-invasive monitor.<br>3. central line in over 18yrs, whereas younger age group had a 16gauge cannula placed in the left external jugular vein<br>4. patient positioning in lateral position<br>5. After positioning a paravertebral block at the T4 level was given with 0.25% Bupivacaine (0.75ml/kg) for intra and post operative analgesia.<br>6. Anaesthesia was maintained with oxygen, air and isoflurane<br>7. extubation at the end of surgery after reversing neuromuscular blockade with neostigmine injection<br>8. Standardised surgical technique: posterolateral thoracotomy and chest was entered through the bed of the forth rib. The mediastinal pleural was dissected and PDA was identified. This was dissected out and after reduction of the arterial pressure the PDA was ligated using Ethibond No.2. The mediastinal pleura was approximated and chest closed in the routine fashion over an intercostal drain.	1. Arterial line and intercostal drain removal after 6 hours<br>2. Patient moved to HDU at 6 hours to be cared for by family<br>3. Discharge on POD2<br>4. Postoperative education<br>5. Postoperative follow-up at 1 week<br>6. Standardised cardiac monitoring along with arterial blood gases and chest x-ray
Baluku et al, 2020 [4]	adapted	1. Counselling and ERAS education<br>2. No fasting to both solids and liquids<br>3. Prophylactic antibiotics<br>4. Prophylaxis against PONV (8 mg of IV dexamethasone)<br>5. Prophylaxis against pulmonary aspiration (100 mg of IV ranitidine and 10 mg of IV metoclopramide)	1. Single-shot spinal with 10‚Äì12.5 mg of plain hyperbaric bupivacaine and 100 mcg of preservative-free ITM<br>2. Restrictive fluid administration to ensure normovolemia<br>3. Treatment of hypotension with a continuous adrenaline infusion of 100 mcg in 500 mL of lactated Ringer’s solution and 6-mg boluses of ephedrine<br>4. Prevention of hypothermia with warm IV fluids and warm clothing cover<br>5. Local wound infiltration analgesia with 2 mg/kg of bupivacaine<br>6. Rectal diclofenac (100 mg)<br>7. Rectal misoprostol (400 Œºg)	1. Carbohydrate drink within 1 h<br>2. Cessation of IV fluids within 1 h<br>3. Early breastfeeding, within 30 min<br>4. Analgesia with oral single fixed-dose combination of 400 mg of ibuprofen and 500 mg of acetaminophen every 8 h. Breakthrough pain was treated with 25 mg of IV pethidine.<br>5. Early mobilization at 8 h<br>6. Early urethral catheter removal at 6 h<br>7. Oral antibiotics (850 mg of amoxicillin-clavulanate q12h and 500 mg of metronidazole every 8 h)<br>8. Discharge based on clinical criteria<br>9. Given emergency contact telephone number to report and health concerns at home
Bansal et al, 2020 [5]	adapted	1. Avoidance of any oral or per-rectal mechanical bowel preparation<br>2. Allowance of solid food till 8 h before surgery<br>3. Injectable third-generation cephalosporin (ceftriaxone), aminoglycoside (amikacin) and metronidazole started one night before surgery.	None	1. Avoidance of long-acting intravenous opioids<br>2. Early removal of resection site drainage<br>3. Early removal of the nasogastric tube by the POD1 morning<br>4. Use of gum chewing postoperatively thrice a day for 1 h each from POD1<br>5. Multimodal antiemetic prophylaxis using metoclopramide 10 mg twice daily and ondansetron 4 mg thrice a day till taking full oral diet<br>6. Multimodal analgesia (epidural opioids, intravenous and oral nonopioid drugs)<br>7. Early enteral feeding<br>8. Early obligatory ambulation

				9. Standardised antibiotic management<br>10. Criteria-led discharge
Chaudhary et al, 2015 [6]	adapted	1. No bowel preparation<br>2. Overnight fasting<br>3. A single dose of ertapenem before induction of surgery	1. single surgeon operation<br>2. classical PD, if duct was not visualised end-to-side pancreatojejunostomy<br>3. drain placement on a case-to-case basis	1. Early removal of nasogastric tube (POD1)<br>2. Early oral feeding (sips POD1, progress on subsequent days)<br>3. Thromboprophylaxis (low molecular weight heparin and compression stockings)<br>4. Prokinetics (POD1 to POD5)<br>5. Early removal of epidural catheter (POD2)<br>6. Early removal of urinary catheter (POD2)<br>7. Stop antibiotics (POD2)<br>8. Early ambulation (start POD2)<br>9. Prepare for discharge (POD5)<br>10. No total parenteral nutrition<br>11. Chest physiotherapy<br>12. Criteria-led discharge
Iyer & Kareem, 2019 [7]	adapted	1. Complete information about the protocol was given and consent taken<br>2. Reviewed in the outpatient department<br>3. Minimal starvation period<br>4. Avoidance of mechanical bowel preparation<br>5. A 100 g oral carbohydrate drink was given to the patient to be consumed along with other clear liquids up to 6 h before surgery<br>6. Basic blood investigations on admission	1. Elective use of abdominal drains, urinary catheters and nasogastric decompression, only when absolutely essential<br>2. Strictly avoiding overhydration	1. Early enforced mobilization<br>2. Early enteral nutrition<br>3. Early removal of all drains, catheters, and tubes
Jain et al, 2015 [8]	adapted	1. Optimisation of patients prior to surgery<br>2. Detailed clinical evaluation performed, comorbid conditions were recorded, and laboratory investigations requested<br>3. A member of the anesthesia team evaluated the patient in the emergency area to assist with optimization of the patient. Subspecialty consult, was sought if requested by the internal medicine or anesthesia teams.<br>4. Patients received Cefuroxime (1.5g iv) followed by 750mg iv every 8 hours for 48 hours in the postoperative phase.<br>5. Other antibiotics were added as advised by the physician team for chest infection, urinary tract infection, and so on.	1. Standard surgical procedure: Internal fixation with cannulated screws was performed for undisplaced and valgus impacted fractures as well as in the frail elderly patients with poor cognitive function (mini mental test<7) or multiple comorbidities who were considered poor can-didates for hip arthroplasty. Patients with displaced femoral neck fractures were treated with cemented modular bipolar arthroplasty<br>2. All surgeries performed under spinal/epidural anaesthesia by 1 of the 6 treating surgeons.<br>3. The choice of approach (modified Hardinge or posterior) depended on the treating surgeon.<br>4. All cases performed or assisted by an attending orthopaedic surgeon	1. Postoperative antibiotics (Cefuroxime 750mg intravenously 8 hourly for 48 hrs)<br>2. Management in the postoperative ward manned by anaesthetic team for 2-4 hours<br>3. Stable patients transferred to orthopaedic ward and those needing active medical management were transferred to high dependency unit for stabilisation<br>4. Daily input from internal medicine<br>5. DVT prophylaxis<br>6. Postoperative X-rays<br>7. Encouraged early mobilisation<br>8. Posterior hip precautions (as required)<br>9. Discharge directly home<br>10. Outpatient follow-up (day 12, at 6 weeks, 3 months, 6 months and then 6 monthly)
Khowaja, 2006 [9]	Not reported	Not reported	Not reported	Not reported

Kulshrestha et al, 2019 [10]	adapted	1. Patient optimization<br>2. Anaesthetic evaluation<br>3. Baseline haematological and biochemical tests<br>4. Next of kin counselling<br>5. Transfer with trauma precautions<br>6. Prevention of over-investigation of patients with non-modifiable risk factors<br>7. Preoperative antibiotic prophylaxis<br>8. Operation within 24 hours of arrival at the hospital. For patients with modifiable risks that require optimization, expeditious management was started in 24 to 48 hours.	1. Minimally invasive surgery<br>2. Antibiotic prophylaxis for urinary catheterization	1. Postoperative antibiotic prophylaxis<br>2. Special care was taken to avoid electrolyte imbal- ance, hypotension, renal insult, deep vein thrombosis, and bedsore.<br>3. Avoided use of narcotics and sedatives<br>4. Aggressive physical therapy protocol<br>5. Thromboprophylaxis (mechanical and chemical)<br>6. Early ambulation<br>7. Criteria-led discharge
Kurmi et al, 2020 [11]	adapted	1. preoperative water soluble contrast enema and/or ileocolonoscopy to demonstrate distal anastomotic integrity<br>2. preadmission counselling by the operating team<br>3. saline feed (500 ml DNS over 2 h) from the distal loop of stoma a day prior to stoma closure to confirm the distal patency and also to act as priming agent (feed) to the distal bowel<br>4. Fasting for solids for 6 h and for liquids (plain water and carbohydrate rich drinks) for 2 h prior to surgery<br>5. preoperative carbohydrate loading with 200 ml apple juice (containing 100 kcal) 6 h and 2 h before surgery<br>6. No premedication	1. no nasogastric tubes, urinary catheter, or abdominal drain<br>2. intraoperative neutral fluid balance (intraoperative restrictive fluid therapy (Plasmalyte), i.e., not exceeding > 1000 ml)<br>3. General anaesthetic with ultrasound-guided unilateral transverse abdominis plane block at the stoma site with ropivacaine 0.25% after patient was anaesthetized<br>4. surgical technique: Circumstomal incision was made, stoma mobilized, and margin refreshened. The stoma was closed by intraperitoneal method, end-to-end anastomosis in a double-layer with delayed absorbable suture. If inadvertent perforation occurred, it was either primarily repaired (if away from the stomal opening) or resected and end-to-end anastomosed if nearby, depending on the discretion of the operating surgeon. The sheath was closed with polypropylene no 1 suture.	1. initiation of oral feed as early as possible after 6 h of surgery (irrespective of the bowel sound)<br>2. early ambulation (sitting, standing, or out of bed after 6 h of surgery)<br>3. visual analog scale (VAS) score for pain was assessed and patients received injectable diclofenac, paracetamol, or fentanyl depending on the score<br>4. discharge on the first or second day of surgery depending on the patient comfort.<br>5. After discharge, the patients were followed up in surgical outpatient clinic on the seventh day, second week, and fourth week of discharge
Kuzmenko et al, 2019 [12]	adapted	1. Informing the patient<br>2. clear fluids and carbohydrate until 2-3h and eating until 6h before surgery<br>3. no bowel preparation	1. used of general anaesthesia with epidural analgesia<br>2. infusion therapy in zero balance with crystalloids (4ml/kg/hr)<br>3. hemotransfusion with a haemoglobin index below 80g/L<br>4. Installation of abdominal drainage in dependence from the risk of PF (pancreatic fistula) formation according to the International Study Group of Pancreatic surgery (ISGPF) classification<br>5. Antibiotic prophylaxis with the antibiotic cephalosporin of the II generation 30-60minutes before the operation and again after 4-6hours.<br>6. standardised surgical technique with microjejunostomy	1. transferred to ICU<br>2. mobilised with 18-24hrs<br>3. During the first 18-24hrs NGT was removed after X-ray control of the passage from the stomach<br>4. oral nutrition started on Day 1. up to 250ml on day 1, up to 500ml on day 2, up to 1L on day 3. patients started taking solid food on day 4<br>5. Infusion therapy with crystalloids was carried out in the zero-balance regime and stopped on Day 4.<br>6. Determination of the concentration of drainage amylase was carried out on PoD1 and PoD3.<br>7. Drainages were removed on 4-6 PoD in case the absence of secretions<br>8. Thromboprophylaxis consisted in the application of compression of the lower extremities by elastic knitwear and the induction of low-molecular heprain drugs.

				9. Somatostatin analogues were used only at high risk PF formation for 3 days<br>10. nonsteroidal antiinflammatory drugs and insufflation of lidocaine in epidural catheter were used for analgesia
Mahendran et al, 2019 [13]	adapted	1. Preoperative investigations (CT and upper gastrointestinal endoscopy and biopsy in all, endoscopic ultrasound-guided biopsy in some)<br>2. Preoperative biliary drainage was not performed as a routine<br>3. Preoperative chest physiotherapy<br>4. Clear liquids until 4 hrs before surgery<br>5. No mechanical bowel preparation<br>6. Antianxiety medication given<br>7. Preoperative antibiotics	1. Epidural catheter, central line and Foleys catheter<br>2. Sequential compression device<br>3. Hypotensive anesthesia and restrictive fluid policy<br>4. Somatostatin analogues (octreotide)<br>5. Blood transfusion was not preferred if hemoglobin > 8 mg/dL	1. Early and progressive mobilisation (sitting in bed POD0, ambulation at least 3 times POD1, ambulation 6 times POD2)<br>2. Early oral intake (Oral sips clear liquids POD1, clear liquid diet POD2, semisolid diet POD3, normal diet POD5)<br>3. Chest physiotherapy<br>4. IV fluid support based on intake and stopped on POD3 if intake adequate<br>5. Removal of central line, urinary catheter and epidural catheter POD2<br>6. IV analgesia decreased POD3 and stopped POD4<br>7. Criteria-led drain removal POD4<br>8. All medication stopped except oral proton pump inhibitors, metoclopramide, multivitamin POD4<br>9. IV access removed POD5<br>10. Plan for discharge POD6<br>11. Criteria-led discharge
Mangukia et al, 2019 [14]	adapted	1. Patient education and preparation<br>2. Patient screened for potential comorbidities<br>3. All medications were stopped 3 days preoperatively in elective cases, except for aspirin, metoprolol (titrated for a target heart rate of 60–70 beatsmin1), omeprazole, atorvastatin, and oral hypoglycemic drugs or insulin.<br>4. LMWH to bridge for urgent surgery<br>5. ECG<br>6. CXR<br>7. ultrasound of the abdomen<br>8. random blood sugar level<br>9. complete blood count, liver, kidney, and pulmonary function tests, bleeding profile, serum electrolytes, and tests for viral markers.<br>10. Active smokers with mild to moderate chronic obstructive pulmonary disease (COPD) on bronchodilator treatment, who were candidates for elective surgery, were asked to stop smoking for at least 6 weeks.<br>11. Left radial duplex Doppler in all cases selected for radial artery harvest.<br>12. Mupirocin 2% nasal ointment was used twice a day for 2 days preoperatively	1. Newer anesthetic and analgesics with the aim of reducing surgical stress responses, pain, and discomfort<br>2. Off-pump coronary artery bypass conducted using usual surgical standard of hospital<br>3. No elective use of pacing wires<br>4. Chest tubes inserted from the rectus fascia<br>5. Antibiotic prophylaxis<br>6. Balanced anesthesia was given (moderate opioids, hypnotics, and muscle relaxants) with standard endotracheal intubation<br>7. Invasive monitoring (arterial line, central line, Swan-Ganz catheter, transoesophageal echocardiography, femoral line for patients with EF<20%)	1. Fast-track extubation within 4 hours<br>2. Intensive care unit admission postoperatively<br>3. Standardised postoperative investigations<br>4. Standardised postoperative medication management<br>5. Minimal use of opioid analgesia after day 2<br>6. Aggressive postoperative rehabilitation<br>7. Early enteral nutrition<br>8. Early ambulation<br>9. Optimized management of tubes and invasive lines<br>10. Daily telephone follow-up for 1 week after discharge<br>11. Benzodiazepines for sleep and anxiety<br>12. Criteria-led transfer from ICU to ward<br>13. Sunlight exposure in the morning on POD2<br>14. Criteria-led discharge<br>15. Detailed postoperative instruction manual provided to patients<br>16. Wound examination POD7 and at one 1 month<br>17. ECHO at follow-up as required

		13. Pulmonary function tests were avoided in patients with > 80% left main disease, recent unstable angina, and electrocardiogram changes.<br>14. Patients encouraged to undertake the recommended physiotherapy exercises for at least 3 days before scheduled surgery, to ensure familiarity and allow comparison between pre- and postoperative exercise endurance.<br>15. ECHO<br>16. Preoperative scrub baths		
Nanavati & Prabhakar, 2014 [15]	adapted	1. Minimal starvation<br>2. Oral carbohydrate drink<br>3. Pre-operative antibiotic, acid suppression and pro-kinetic<br>4. No mechanical bowel preparation<br>5. Patient counselling	1. Elective use of nasogastric decompression, urinary catheterisation and abdominal drainage<br>2. No over-hydration<br>3. Minimal tissue handling<br>4. Minimise operative time<br>5. Minimally invasive surgery and-sewn technique of anastomosis<br>6. Peri-incisional local anaesthetic	1. Early removal of all drains and tubes if inserted<br>2. Early enteral nutrition<br>3. Early enforced ambulation<br>4. Pre-emptive and adequate analgesia<br>5. Ensure follow-up after discharge<br>6. Criteria-led discharge
Nanavati & Prabhakar, 2015 [16]	adapted	1. Provide complete information about the protocol and take an informed consent<br>2. Advice given regarding exercise, smoking and alcohol cessation<br>3. Optimise any pre-existing co-morbidity<br>4. Minimal starvation (6 h for solids and 2 h for liquids)<br>5. 100 g oral carbohydrate drink<br>6. Avoid mechanical bowel preparation<br>7. Pre-operative antibiotic	1. Epidural anaesthesia (0.125 %bupivacaine, continuous infusion) along with spinal or general anaesthesia<br>2. Arterial/central lines inserted only if unavoidable<br>3. Strictly avoiding over-hydration<br>4. Maintain optimal oxygenation<br>5. Avoid hypothermia<br>6. Minimal tissue handling<br>7. Elective use of nasogastric tubes, abdominal drains and urinary catheters	1. Maintain supplemental oxygen<br>2. Strict post-operative nausea and vomiting prophylaxis<br>3. Early enforced mobilisation<br>4. Early enteral nutrition<br>5. Removal ofepidural catheter by day 2<br>6. Ensuring adequate analgesia after epidural catheter removal<br>7. Early removal of all tubes, drains and catheters<br>8. Ensure 30-day follow-up including; phone call at 48 h, seventh day clinic visit and any emergency visit
Pal et al, 2003 [17]	designed de novo	1. Voiding prior to surgery to minimise use of urinary catheter<br>2. Standardised preoperative investigations<br>3. Blood cross-matching decisions to only be taken by a senior member of the clinical team<br>4. Admit low-risk patients on the day of surgery (American Society of Anesthesiologists grade I & II)_	1. Use single-lumen Ryle's type tube for gastric decompression<br>2. Stop use of Opsite drapes<br>3. Use of reusable metal catheter<br>4. Standardisation of suture material<br>5. Selective use of operative cholangiogram<br>6. Single-dose antibiotic prophylaxis<br>7. All IV fluid were administered from 1 Liter bottles	1. Standardised oral intake<br>2. Standardised mobilization
Pandit et al, 2019 [18]	adapted	1. Preoperative counselling<br>2. Preoperative carbohydrate loading, with clear liquid of 50 gm, and 200 ml carbohydrate-rich drink on the evening, as well as 3 hours before surgery<br>3. Preoperative biliary drainage only in cholangitis or severe malnutrition	1. Mid-thoracic epidural anesthesia<br>2. Perioperative goal-directed IV fluid therapy using hemodynamic monitoring via arterial line	1. Early removal of nasogastric tube (Day 1);<br>2. Early oral feeds (sips on first day, clear liquids on second day and gradually progressed to solid diet by day 6)<br>3. Early Foley's catheter removal (Day 3)<br>4. Early mobilization (on bed active limb movement in day 1, bedside sitting/standing on day 2 and assisted walking on day 3)

				5. Restricted intravenous fluids in postoperative periods with permissive relative oliguria (0.4 to 0.5 ml/ kg/hr);<br>6. Early intra-abdominal drain removal, day 3 for firm pancreas and day 5 for soft pancreas and the drain amylase value less than 3-fold of serum level;<br>7. Non-narcotic analgesia
Pillai et al, 2014 [19]	adapted	1. Preoperative information given to patient, including daily milestones	1. Thoracic epidural inserted for analgesia	1. Multimodal analgesia<br>2. Criteria-led removal of nasogastric tube on POD1<br>3. Mobilisation started POD1 and progressively increased<br>4. Start feeding through feeding jejunostomy POD1<br>5. Urinary catheters removed POD2<br>6. Clear oral liquids POD3<br>7. Criteria-led removal of drainage tubes POD3<br>8. Soft solid diet POD4<br>9. Dietary increase on daily basis from POD5<br>10. Epidural catheter removal POD5<br>11. Nausea and vomiting prophylaxis (metacloperamide)<br>12. Criteria-led discharge<br>13. Post-operative high-dependency unit nursing and criteria-led discharge to ward
Pirzada et al, 2017 [20]	adapted	1) Patients were counselled about modified ERAS protocol<br>2) fasting of less than 6 hours<br>3) fluid and carbohydrate loading with Glaxose-D (glucose juice) 6 hours before surgery<br>4) antibiotic prophylaxis.<br>5) No premedication<br>6) no bowel prep	1) short acting anaesthetic agents like sevoflurane/atracurium were used,<br>2) bowel anastomosis were performed by hand-sewn method using two-layer anastomosis, first-layer continuous suturing full thickness technique and second- layer interrupted seromuscular sutures using vicryl # 4/0 suture material.<br>3) Drain in abdominal cavity, urinary catheter and nasogastric tube were avoided.<br>4) avoidance of salt and water overload<br>5) avoidance of hypothermia<br>6) All surgeries were performed by senior surgeons.	1. Prevention of nausea and vomiting<br>2. Paracetamol was used for pain management<br>3. Patients were asked to mobilise 6 hours after the surgery<br>4. Oral sips (15ml/hour) were started. Patients were continued to free clear liquids within 24 hours if tolerated orally and then to semi-solid diet after 24 hours of surgery.
Quader et al, 2010 [21]	adapted	1. Pre-operative counseling<br>2. Pre-operative investigations and pre-anesthetic check-up completed before hospital admission.<br>3. All patients were admitted at least 1 day prior to surgery.<br>4. Patients were provided with the date of surgery and the telephone number of a senior resident to contact in case of confusion or delay/failure to arrive on the said date.	1. A rapid-recovery protocol emphasizing reduced CPB time.<br>2. Standardized anaethetic technique using short-acting anesthetic/analgesic agents and monitoring of lead II ECG, invasive blood pressure, central venous pressure, pulse oximetry, end-tidal CO2, nasopharyngeal temperature, urine output, ACT and intermittent blood gas analysis in all patients<br>3. Perioperative administration of corticosteroids and tranexamic acid<br>4. Standardized surgical procedures.<br>5. Minimal use of blood products.<br>6. Intra-operative transoesophageal echocardiography (TOE) was used if indicated.<br>7. Full rewarming and maintaining normothermia.	1. Transfer to ICU immediately post-operatively.<br>2. A protocol for early extubation (within 6 hours) in the presence of a senior team member using specified criteria<br>3. Temporary pacing wires removed on the morning of 1st post operative day (POD) if rhythm stable.<br>4. Early removal of drains/invasive lines (on POD1)<br>5. Discharge from ICU to ward within 24 hours according to criteria<br>6. Aggressive diuresis applied to all patients.<br>7. Early ambulation and intense physiotherapy.<br>8. Early resumption of enteral feeds.<br>9. Judicious use of antibiotics and analgesics.<br>10. GI prophylaxis.<br>11. Discharge after 72 hours if met discharge criteria (walking without assistance, had return of normal bowel function, with well controlled pain and willingness to go home)

				12. Patients were followed up over telephone by a senior resident at 12 h, 24 h and 3 days after discharge.<br>13. Clinical out-patient follow-up was performed at 7 and 30 days after discharge.<br>14. Low-dose diuretics
Sahoo et al, 2014 [22]	adapted	1. Explain the patients and their relatives about ERAS and take informed consent<br>2. Malnourished patients are given hyperproteinic supplements during the week before surgery<br>3. No bowel preparation<br>4. Normal breakfast, lunch and dinner the day before surgery - If cannot tolerate solid food, given liquid diet with high protein and carbohydrates<br>5. Two hours before surgery: provision of high carbohydrate liquid diet<br>6. Antibiotic prophylaxis	1. No drains and no nasogastric tube<br>2. Maintenance of 02/air FiO2 >80%<br>3. Routine monitoring (arterial/central catheter if unavoidable)<br>4. Fluid maintenance with Hartmanns<br>5. Protocolised blood pressure management (MAP >65mmHg)<br>6. Liquid heater and heating blanket<br>7. Standardised surgical technique<br>8. Antibiotic prophylaxis<br>9. Masks with high O2 flow for 2 hrs independent of saturation	1. Protocolised management in recovery room<br>2. Progressive mobilisation from sitting in chair for 2 h (day of surgery), seated at least 6 h (POD1) to mobilisation on demand (POD2)<br>3. Progressing nutrition from liquid diet including high calorie dietary preparation (day of surgery), increasing amount which includes high protein/high calorie diet (POD1) to soft/normal diet (POD2)<br>4. Multimodal analgesia - Paracetamol from day of surgery and NSAIDs started POD2<br>5. Suspend IV fluids on POD1 (if tolerated)<br>6. Criteria-led discharge assessed daily from POD1<br>7. Remove urinary catheter (POD2)<br>8. Telephone monitoring for 48 h<br>9. First out-patient visit 10-14 days after surgery<br>10. Minimum diuresis<br>11. Suspect epidural analgesia on POD2
Sanad et al, 2019 [23]	adapted	1) Patient counselling and education<br>2) Preoperative control of chronic disease (cardiac function, pulmonary function, blood pressure and diabetes)<br>3) Serum Albumin level was maintained greater than 3.5g/dl.<br>4) Preoperative prophylaxis against thrombosis, infection and nausea and vomiting was established<br>5) Fasting at the most for 6hours preoperatively for food and 2 hours for clear liquids. Advised to have plentiful fluid intake before surgery<br>6) Preoperative carbohydrate -rich drinks were used to reduce the effect of fasting<br>7) rectal enemas and mechanical bowel preparations were avoided	1. Minimal access surgery<br>2. Combined general anasthesia with lumbar epidural analgesia.<br>3. Minimal crystalloid administration, with preferential use of colloids and ephedrine<br>4. No use of drains, tubes or catheters	1) Postoperatively patients were advised to use chewing gums, laxatives and drink clear liquids upon awakening from anaesthesia.<br>2) To eat a general diet when begin walking. Intake of protein and energy rich nutritional supplements was advised.<br>3) management of postoperative nausea and vomiting were guaranteed with the use of multimodal approach by 2 or more antiemetics in combination.<br>4) Fluid management aimed to maintain perioperative euvolaemia by minimising crystalloid administration, increasing the use of colloids and the use of vasopressors (ephedrine) in place of crystalloid for treatment of hypotension in a euvolaemic patient<br>5) Pain management: use of opiod-free drugs. combining regional anesthetic techniques with multimodal pharmacologic pain management instead of opioids - consisted of the use of two pain killers with different mode of action as NSAIDs, paracetamol or acetaminophen<br>6) Urinary catheters were removed as soon as possible.<br>7) Early mobilisation: patients got out of bed a minimum of 2hours on the day of surgery<br>8) Followup visit at outpatient clinic at day 7 and day 14 postop
Shah et al, 2016 [24]	adapted	1. Obtain informed consent.<br>2. Educate on fast track rehabilitation program<br>3. Preoperative heparin 5000 units s/c	1. Prescribe perioperative antibiotics<br>2. Obtain central access as per anaesthesia assessment.<br>3. Fix nasogastric tube and intra-abdominal drain<br>4. No prokinetic drug or octreotide was given routinely	1. Administer intravenous patient controlled analgesia with morphine plus intravenous paracetamol or NSAID's, PPI<br>2. Ensure first night in ICU or intermediate care unit<br>3. Remove nasogastric tube if drainage amount <300 mL (POD1)

				4. Remove Foley catheter; allow sips of water (<30 mL/hour) (POD1)<br>5. Achieve postoperative pain control by stepwise dose reduction and transition to non-opoid medication or paracetamol (0.5-1 gm/day QDS) (POD1)<br>6. Continue I/V fluid administration till adequate oral fluid intake (POD1) and then reduce until discontinuation on POD4)<br>7. Progressive increase in oral intake, with commencement of clear liquid diet (POD2), introduction of light mashed diet (POD3) and normal oral diet from POD4.<br>8. Progressively enhance patient mobilization: POD 2 >2 hours out of bed and POD3 >4 hours out of bed.<br>9. Remove drain when there is no pancreatic or biliary fistula or when drain amount is <200 mL/day (POD5/6)<br>10. Seek medical or radiation oncology consultation, if necessary (POD5/6)<br>11. Criteria-led discharge POD6/7 (a) Absence of fever (<37.5 °C) for more than 48 hours, (b) Adequate control of pain with oral analgesics, (c) Ability to take solid food, (d) History of passage of stools, (e) Adequate mobilization<br>12. Prescription for discharge medications (PPI, pancreatic enzyme, analgesic)<br>13. Follow-up appointment 2 weeks after discharge<br>14. Thromboprophylaxis with low molecular weight heparin
Shetiwy et al, 2017 [25]	adapted	1. Preoperative counselling by ERAS team (about the proposed fast track care plan and about the daily chores, milestones and expectations regarding the procedure<br>2. Carbohydrate-rich drinks on the day before surgery<br>3. Drinking is encouraged until 4 hours preoperatively (morning of surgery)<br>4. Preoperative mechanical bowel preparation only for rectal/rectosigmoid malignancy<br>5. Mandatory prophylaxis against thromboembolism (special deterrent stockings and a single dose of low molecular weight Heparin [LMWH] in the evening before surgery - 6:00 PM)	1. Fluid management - Avoidance of sodium/fluid overload<br>2. Assisted laparoscopic surgery with small transverse incision preferred over longitudinal incisions<br>3. Mandatory intraoperative warming of patients and IV fluids<br>4. Standardized anaesthetic protocol.<br>5. No pre-anaesthetic medications allowed.<br>6. Nasogastric tubes (NGTs), urinary catheters and intra-abdominal drains were routinely inserted<br>7. All patients, unless they had refused, had low thoracic epidural catheters (T10,Äì12) inserted for postoperative pain control.	1. NG tube removal on the day of surgery (POD 0) except for patients with PONV<br>2. Oral sips within 24 hours of surgery and resume full diet on POD 3 with IV fluid restricted to a minimum<br>3. Forcing patients to get out of bed for 2 hours postoperatively (on POD 0) and on the morning of POD 1, gradually increased to 6 hours by the time of discharge.<br>4. Opiates not allowed in epidural analgesia<br>5. Epidurals for 48 hours only<br>6. Early oral analgesia with regular doses (acetaminophen + NSAIDs) after 48 hours<br>7. Stimulation of gut motility allowed with oral/rectal laxatives<br>8. Warming blankets used for 2 hours postoperatively.<br>9. Immediate postoperative recovery area (ICU, HDU, ward) decided based on vital signs and pre-existing comorbidities<br>10. Discharge once criteria met (no complications at the time of discharge, tolerance to food intake (solid diet) with normalization of gastrointestinal tract functions, successful oral analgesia, independent mobilization, and acceptance (consent) of hospital discharge)<br>11. Follow-up within 1 week(Patients were readmitted when they presented on their follow update or sooner with a surgical complication that could not be managed in the outpatient clinic, i.e., persistent PONV, severe urinary tract infection (UTI),

				postoperative ileus, and intra-abdominal collection on follow-up ultrasound)<br>12. Urinary catheters were usually removed on POD 0 unless ureteric/bladder injury had occurred (continuous drainage for 14 days).<br>13. After full oral intake had been maintained, drains were removed
Shrikhande et al, 2013 [26]	Not reported	1. CT abdomen and pelvis<br>2. patient center management<br>3. case managed by the gastrointestinal disease management group	1. Pancreatico-enteric anastomosis was performed as an end to side, duct to mucosa PJ in two layers with fine PDS sutures; Pylorus-preserving PD (PPPD)<br>2. Prophylactic antibiotics<br>3. Octreotide<br>4. A single portex drain (in the Morrison's pouch) preferentially over two drains<br>5. A nasojejunal tube was routinely placed intraoperatively	1. Drain fluid amylase was checked on POD3 and POD7<br>2. Abdominal drains were removed if drain amylase POD 7 was within normal limits.<br>3. Continued antibiotic prophylaxis (3 days)<br>4. Continued octreotide (7 days)<br>5. All patients managed in ICU for 12-24hrs<br>6. Step down unit management after ICU for 24-48hrs<br>7. Surgical ward following step down unit management<br>8. 30-days follow-up for complication
Vashistha et al, 2018 [27]	Designed de novo	"1. Examination and initial evaluation in the emergency room<br>2. Transfer to surgical intensive care unit for preoperative stabilization and work up<br>3. Cultures and empirical antibiotic therapy<br>4. Abdominal CT unless pneumoperitoneum on Xray<br>5. All preoperative imaging was reported by radiologist(s) experienced in GI radiology	1. Operation conducted by consultants experienced in advanced digestive tract surgery	1. Postotoperative management in surgical intensive care unit<br>2. Adequate pain relief<br>3. Early extubation (possibly within first 24 h after satisfying clinical criteria<br>4. Vigorous chest physiotherapy<br>5. Early ambulation initiated preferably within first 24 h of surgery.<br>6. Deep vein thrombosis prophylaxis initiated preferably within first 24 h of surgery<br>7. Early initiation of enteral feeding.<br>8. Total parenteral nutrition was sparingly utilized<br>9. Postoperative antibiotics were continued for a minimum 7 days<br>10. De-escalation/switch over was made to culture-specific antibiotics<br>11. Discharge from surgical ICU once haemodynamically stable and oral feeding has commenced<br>12. All postoperative investigations were done 'on-demand' basis only

Table 2: Strategies used for care pathway implementation

(The ERIC strategy is named with the associated article specific data in brackets. Where appropriate, direct quotations have been used)

Source	Strategies used for care pathway implementation
Agarwal et al, 2018 [1]	1. Conduct educational meetings (A multidisciplinary team consisting of gastrointestinal surgeons, anaesthetists, trainees, nurse, physiotherapists, and nutritionists was trained regarding en- hanced recovery elements during the perioperative period)<br>2. Conduct ongoing training (Following the initial implementation, periodic reinforcements ofER concepts were done by lectures and one-to-one basis as needed.)

	3. Develop and organize quality monitoring systems (Developed to monitor compliance to all elements of the pathway)<br>4. Develop educational materials (Smartphones were used for flashing alerts and resource material.)<br>5. Distribute educational materials (Smartphones were used for flashing alerts and resource material.)<br>6. Prepare patients/consumers to be active participants (Preoperative counselling as part of the pathway)<br>7. Promote adaptability (Some recommendations were not implemented for various reasons such as the following: (a) preoperative immune-nutrition and intraoperative goal-directed fluid therapy were not feasible financially; (b) sur- geons preferred to administer somatostatin analogues to majority of the patients until May 2016 thereafter it was discontinued; and (c) being a referral centre, preoperative selective biliary drainage was not feasible, as patients presented to the hospital after the drainage procedure)<br>8. Remind clinicians (Smartphones were used for flashing alerts and resource material.)<br>9. Use advisory boards and workgroups (Implementation ofER programme involved building a team and circumventing challenges within each discipline.)
Ahmed et al, 2010 [2]	1. Prepare patients/consumers to be active participants (Preoperative education as part of the pathway)
Akhtar et al, 2000 [3]	1. Prepare patients/consumers to be active participants (patient education and preparation was part of the pathway)<br>2. Stage implementation scale up (the pathway method has been expanded to surgery for other congenital and acquired heart conditions)<br>3. Promote adaptability (modified the pathway for patients and families who could not reach the hospital in the morning at the required time)<br>4. Create new clinical teams (the surgeon planner, the clinical nurse co-ordinator, physician and nurse implementers and family were actively involved in the successful use of the pathways)
Baluku et al, 2020 [4]	1. Promote adaptability (We did not provide a standard carbohydrate drink for early oral feeding; rather, we allowed mothers to drink anything that contained carbohydrates or sugar AND Post discharge follow-up was done via telephone because we do not have community midwives in our health system))<br>2. Prepare patients/consumers to be active participants (Counselling and ERAS patient education)<br>3. Develop academic partnerships (scholarship awarded to first author from Massachusetts General Hospital and Mbarara University Research Training Institute (MURTI) for the training program in manuscript writing)<br>4. Work with educational institutions (scholarship awarded to first author from Massachusetts General Hospital and Mbarara University Research Training Institute (MURTI) for the training program in manuscript writing)
Bansal et al, 2020 [5]	1. Promote adaptability (We did not use clear carbohydrate-rich drinks in the preoperative period (due to nonavailability))
Chaudhary et al, 2015 [6]	1. Purposely reexamine the implementation (We analyzed our experience with patients undergoing PD with a predefined enhanced recovery protocol, to determine the earliest postoperative day on which most of our patients could be safely discharged and to identify preoperative factors, if any, which could possibly lead to prolonged hospital stay despite the absence of postoperative complications. The rationale was to determine whether such protocols can be universally applied to all patients and, if not, to determine whether changes can be implemented to the existing protocols so as to tailor them to specific patient subsets.)
Iyer & Kareem, 2019 [7]	1. Prepare patients/consumers to be active participants (Preoperative counseling as part of the pathway)
Jain et al, 2015 [8]	1. Promote adaptability (For displaced femoral neck fractures, we chose a cemented modular bipolar prosthe- sis, which has been widely used in

	India for more than 10 years.8,9,10,18 Cemented bipolar prostheses of good quality are available from local manufacturers (eg, Ormed, INOR, etc) and are quite cost-effective as compared to THA. Moreover, there is a lack of good-quality cementless stems or THA systems manufactured locally at cheaper costs.)<br>2. Prepare patients/consumers to be active participants<br>3. Build a coalition (The Departments of Orthopedics and Internal Medicine worked together to formulate a treatment algorithm for geriatric hip fractures for our institution).<br>4. Promote network weaving (The Departments of Anesthesia, Physical therapy, and Nursing were sensitized to the special needs and protocols of this program.)
Khowaja, 2006 [9]	1. Stage implementation scale up ("the researcher saw the value of testing this concept of patient care in her work setting and, in the event of supportive findings, changing the model of nursing practice at Aga Khan University Hospital (AKUH) from the traditional to the multidisciplinary approach" and by use of a pilot "Considering the size of the project and the number of variables required to be measured, the data collection plan was thoroughly outlined, and all suggestions outlined during pilot phase of study were rigorously followed to ensure proper collection, recording and storing of data")<br>2. Facilitation ("The researcher also had ongoing dialogue with AKUH unit staff to explore any ambiguities in design and content of the clinical pathway and to ensure the successful implementation of the pathway")<br>3. Develop and implement tools for quality monitoring (All the process and outcome measures were developed and piloted in collaboration with stakeholders, and "the data collection plan was thoroughly outlined, and all suggestions outlined during pilot phase of study were rigorously followed to ensure proper collection, recording and storing of data")<br>4. Develop and organize quality monitoring systems (All the process and outcome measures were developed and piloted in collaboration with stakeholders, and "the data collection plan was thoroughly outlined, and all suggestions outlined during pilot phase of study were rigorously followed to ensure proper collection, recording and storing of data" and Construct validity was used for instruments of process and outcome measurement, which were variance-tracking instruments and the clinical pathway. For variance tracking instruments, content validity was also used as it contained all the important aspects of patient care. This instrument consisted of 57 items out of which 35 items were related to variances in patient care, 11 items related to monitoring clinical indicators, and 11 items related to monitoring financial variances")<br>5. Involve patients/consumers and family members (Once the goals are set, the nurse and patient collaborate to formulate the means by which the goals can be attained)
Kulshrestha et al, 2019 [10]	1. Prepare patients/consumers to be active participants (Preoperative counseling as part of the pathway)<br>2. Build a coalition (the radiology department, an orthopaedic surgeon, a physician, an anaesthetist, an operating room matron, physiothera- pists, and porting services. Nursing and emergency room staff was well acquainted with special needs and protocols of GHFP.)<br>3. Develop and implement tools for quality monitoring (We initiated the Geriatric Hip Fracture Registry, in which data that would help us study the effect of the program was recorded)<br>4. Facilitation (An orthopaedic matron (PS) was designated as the dedicated trauma coordinator for fast tracking patients with hip fractures to surgery)<br>5. Revise professional roles (An orthopaedic matron (PS) was designated as the dedicated trauma coordinator for fast tracking patients with hip fractures to surgery.)<br>6. Involve patients/consumers and family members (To ensure rapid discharge and early rehabilitation, we started NOK and caretaker counselling from admission. The aim was to allay all anxiety of the NOK and involve them in the care of the patient from the beginning.)

Kurmi et al, 2020 [11]	None
Kuzmenko et al, 2019 [12]	1. Prepare patients/consumers to be active participants (Preoperative management of patients under the ERAS includes: informing the patient)
Mahendran et al, 2019 [13]	None
Mangukia et al, 2019 [14]	1. Create new clinical teams (The FT team included cardiac anesthetists, physicians, surgeons, intensivists, and physiotherapists.)<br>2. Distribute educational materials (A detailed postoperative instruction manual describing precautions and instructions was provided and discussed with every patient (printed in the local language).<br>3. Prepare patients/consumers to be active participants (Preoperative counseling as part of the pathway)<br>4. Promote adaptability (As our experience with FT grew, we started using benzodiazepines on day 1 more generously as a sleep aid as well as for anxiety.)
Nanavati & Prabhakar, 2014 [15]	1. Prepare patients/consumers to be active participants (Preoperative counseling as part of the pathway)<br>2. Promote adaptability (Due to financial constraints of the patients and restricted facilities at our government hospital, it was not possible to perform as- sessment of serum markers like pre-albumin and C-reactive protein, but rather we had to use markers like albumin which were available to us.)
Nanavati & Prabhakar, 2015 [16]	1. Prepare patients/consumers to be active participants (Preoperative counseling as part of the pathway)<br>2. Promote adaptability (We did not have the tech- nology available at other leading FTS centres like transesoph- ageal Doppler, etc., to monitor GDFT. The costs involved have been prohibitive at governmental organization like ours.)
Pal et al, 2003 [17]	1. Audit and provide feedback ("We undertook an audit of the in- hospital care process for this surgical procedure using the charge structure set forth in the itemized bill" and "The in-hospital medical care process was defined using the information obtained from the itemized bill. Recommendations addressing changes in the process of care were developed from this review and presented to the general surgery group, which is made up of consultants, residents, and nursing staff")<br>2. Purposefully re-examine the implementation ("The phase II exercise was a focused analysis to determine whether the anticipated changes had taken place")<br>3. Promote adaptability ("An analysis of these 6 categories revealed a number of areas in which changes in practice could be introduced as part of a clinical pathway, with the aim of reducing costs and variability of practice without compromising quality of care")<br>4. Conduct local consensus discussions ("Recommendations addressing changes in the process of care were developed from this review and presented to the general surgery group, which is made up of consultants, residents, and nursing staff. Consensus for change was reached, and the recommendations were used to develop and introduce a clinical pathway for laparoscopic cholecystectomy")
Pandit et al, 2019 [18]	None
Pillai et al, 2014 [19]	1. Prepare patients/consumers to be active participants (Preoperative counseling as part of the pathway)<br>2. Stage implementation scale up (This is a pilot study with 20 patients in each arm before planning a randomized controlled trial.)
Pirzada et al, 2017 [20]	1. Prepare patients/consumers to be active participants ("Pre-operatively, all patients in group B were counselled about modified ERAS protocol")

Quader et al, 2010 [21]	1. Prepare patients/consumers to be active participants ("The entire process of counseling, investigations and pre-anesthetic check-up was completed before hospital admission" and "proper education of patients/parents")
Sahoo et al, 2014 [22]	1. Prepare patients/consumers to be active participants (Preoperative counseling as part of the pathway)
Sanad et al, 2019 [23]	1. Prepare patients/consumers to be active participants (Patient counselling and education is part of the pathway)<br>2. Revise professional roles (In our opinion, the most decisive point was to operate a devoted nurse to supervise the procedure from admission till discharge)<br>3. Build a coalition ("The team was consisted of gynaecological oncologists, anaesthetist, nurse specialist, ward nurse managers, and clinical dieticians")<br>4. Identify and prepare champions (operate a devoted nurse to supervise the procedure from admission till discharge)
Shah et al, 2016 [24]	1. Prepare patients/consumers to be active participants (Preoperative counseling as part of the pathway)
Shetiwy et al, 2017 [25]	1. Promote adaptability ("Kehlet and Wilmore [7] for example proposed a protocol with 15 elements while Wind et al. [1] used 17 elements in their systemic review. Fifteen items were formulated into an enhanced recovery protocol in our study" - i.e. they made their own protocol based on what was appropriate for them/would work in this setting, and "fast track protocols in the literature did not recommend the routine use of abdominal drains following colorectal surgeries except in selective circumstances, such as severe bleeding and difficult dissection ... Although meta-analyses have reported no value of the routine use of drains for colorectal surgeries regarding the postoperative morbidities, we found that they were useful in detecting anastomotic leaks.)<br>2. Prepare patients/consumers to be active participants (" 'Individualized counseling about the proposed fast track care plan and about the daily chores, milestones and expectations regarding the procedure was delivered to patients within the enhanced recovery group.")
Shrikhande et al, 2013 [26]	1. Promote adaptability (Over the three time periods, gradual patient-centric changes were introduced to enable an improvement in the preoperative diagnostic and decision-making algorithm. Also, the pathway was designed de novo)<br>2. Forming a coalition (Most notable amongst these changes was the constitution of the Gastrointestinal Disease Management Group (GI-DMG) from period B onwards. The group is composed of GI and HPB Surgical oncologists, Medical Gastroen- terologists, Radiologists, Intensivists, Interventional Radiologists, Radiation and medical oncologists, and dedicated GI pathologists and clinical co-ordinators who are responsible for carefully moni- toring the patient's preoperative investigations thereby formu- lating a holistic plan for every patient.)<br>3. Create new clinical team (Most notable amongst these changes was the constitution of the Gastrointestinal Disease Management Group (GI-DMG) from period B onwards. The group is composed of GI and HPB Surgical oncologists, Medical Gastroen- terologists, Radiologists, Intensivists, Interventional Radiologists, Radiation and medical oncologists, and dedicated GI pathologists and clinical co-ordinators who are responsible for carefully monitoring the patient's preoperative investigations thereby formu- lating a holistic plan for every patient.)
Vashistha et al, 2018 [27]	None

Table 3: Outcome measures used in care pathway evaluation
(Where possible, direct quotations and nomenclature used in the articles have been listed)

Source	Outcome measures used in care pathway evaluation
Agarwal et al, 2018 [1]	1. Compliance with components of the enhanced recovery pathway<br>2. Postoperative morbidity<br>3. Postoperative mortality<br>4. Length of postoperative stay<br>5. Readmissions<br>6. Surgery-specific complications (clinically relevant postoperative pancreatic fistula, delayed gastric emptying, and post pancreatectomy haemorrhage)<br>7. Functional GI recovery<br>8. Mobilisation
Ahmed et al, 2010 [2]	1. Ventilation time (primary; hours)<br>2. Post-op complications forcing to exclude the case from study (primary)<br>3. Length of ICU stay (primary)<br>4. Mortality (primary)<br>5. Number of patients successfully fast-tracked<br>6. Re-intubation<br>7. Arrhythmia<br>8. Pleural effusion<br>9. Consolidation/lung collapse
Akhtar et al, 2000 [3]	1. Mortality<br>2. Length of stay<br>3. Complications<br>4. Wound infection<br>5. Chest infection<br>6. Recurrent laryngeal palsy<br>7. Reasons for long hospital stay
Baluku et al, 2020 [4]	1. LOHS (primary)<br>2. postoperative pain measured by VAS (measured at 6hrs and every 6 hours until discharge)<br>3. PONV within 24hrs<br>4. pruritus within 24hrs<br>5. urinary retention within 24hrs<br>6. headache within 7days<br>7. wound infection within 30days

	8. puerperal sepsis within 30days<br>9. fever within 30 days<br>10. readmission within 30days<br>11. Fetal apgar score<br>12. Foul smelling lochia
Bansal et al, 2020 [5]	1. Length of stay (primary)<br>2. Time to bowel movement<br>3. Time to flatus and stools<br>4. Time to oral intake<br>5. Time to ambulation<br>6. Time to drain removal<br>7. Postoperative hematological parameters on day 1<br>8. Perioperative complications<br>9. 30-day readmission (reason and incidence)<br>10. Perioperative mortality<br>11. Blood loss<br>12. Operative time<br>13. Intraoperative blood transfusion<br>14. Postoperative blood transfusion
Chaudhary et al, 2015 [6]	1. Length of postoperative hospital stay (primary outcome measure)<br>2. Mortality<br>3. Postoperative pancreatic anastomotic leak/fistula<br>4. Delayed gastric emptying<br>5. Bile leak<br>6. Postpancreatectomy hemorrhage<br>7. Readmission<br>8. Duration of surgery<br>9. Blood loss<br>10. Blood transfusions intraoperatively<br>11. Resurgery/Re-explorations<br>12. Wound infection<br>13. Intra-abdominal collection<br>14. Small bowel obstruction<br>15. Acute pancreatitis<br>16. Ascites<br>17. Gastritis<br>18. Persistent drain output<br>19. Gastrojejunostomy leak

	20. Medical respiratory morbidity<br>21. Medical cardiac morbidity<br>22. Medical neurological morbidity<br>23. Management of readmissions
Iyer & Kareem, 2019 [7]	1. Duration of parenteral analgesia<br>2. Duration of urinary catherization<br>3. Duration of Ryle's tube use<br>4. Duration of abdominal drain use<br>5. Start of feed postoperatively<br>6. Start of ambulation postoperatively<br>7. Time to return of flatus<br>8. Time to return of stools<br>9. Postoperative length of stay<br>10. Time until decision to discharge<br>11. Reasons for delayed in discharge<br>12. Duration of oral analgesia<br>13. Anastomotic leak<br>14. Wound infection
Jain et al, 2015 [8]	1. Time to surgery<br>2. Reason for delay in time to surgery<br>3. Length of hospital stay<br>4. Functional status (ability to mobilise)<br>5. Harris Hip Score (staff administered; considers pain, function, absence of deformity, and range of motion)<br>6. Postoperative electrolyte imbalance<br>7. Delirium<br>8. Posterior hip dislocation<br>9. Periprosthetic fracture<br>10. Surgical wound infection<br>11. Decubitus ulcer<br>12. Acetabular erosion<br>13. Implant loosening<br>14. Mortality<br>15. Management of complications
Khowaja, 2006 [9]	1. Delivery of care - Completion of tasks (i.e. appropriate and complete written physician order; discussion of plan of care to the patients by physicians; complete documentation by nurses in every aspect of patient care; discussion of plan of care with patients and families; appropriate assessment of patient; notification to physician by nurses about patient's condition when required; carrying out physician orders)<br>2. Adherence to time frames for delivering care (i.e. delayed consultation by physician; delayed evaluation; delayed investigation orders written by

	physicians; delayed follow-up and delayed education by physicians to the patients; delayed patient education by nurses; discharge delay due to delivery of medications; timely documentation of discharge notes by nurses; time of discharge orders written by physicians; time of discharge procedure; time patient left hospital; and discharge delays due to family reasons after completion of discharge procedure)<br>3. Electrolyte imbalance<br>4. Constipation<br>5. Phlebitis<br>6. Urinary tract infection<br>7. Haematuria<br>8. Cost (bed charges; attendant fee; surgical fees; anaesthesia charges; pharmacy charges; and medical/surgical supplies charges)<br>9. Patient satisfaction<br>10. Staff satisfaction
Kulshrestha et al, 2019 [10]	1. Readmission<br>2. Mortality<br>3. Ambulatory status and ADL (Parker mobility score)<br>4. Time from injury to admission<br>5. Time from admission to surgery<br>6. Time from injury to surgery<br>7. Surgical site infection<br>8. Prosthetic joint infection<br>9. Dislocation<br>10. Postoperative confusion<br>11. Seizure<br>12. Post-spinal headache<br>13. Cardiac arrest<br>14. Sustained hypotension<br>15. Hyponatraemia<br>16. Blood transfusion<br>17. Length of stay<br>18. Complications at 1 year follow-up
Kurmi et al, 2020 [11]	1. 7-day readmission rate,<br>2. morbidity (Clavien-Dindo classification) rate<br>3. postoperative length of hospital stay<br>4. reoperation<br>5. 30-day mortality rate.<br>6. VAS scores at 12 and 24hrs<br>7. mean time to oral feed initiation<br>8. mean time to passage of flatus<br>9. Surgical site infection

	10. Subacute intestinal obstruction<br>11. Anastomotic leak
Kuzmenko et al, 2019 [12]	1. post operative complications (clavier-dindo classification) including mortality<br>2. occurrence of Delayed gastric emptying<br>3. occurrence of Pancreatic fistula<br>4. time of restoration of oral nutrition<br>5. LOS<br>6. Bleeding from a. gastroduodenalis<br>7. Surgical wound infection<br>8. Perforation of the transverse colon<br>9. The volume of intraoperative infusion therapy
Mahendran et al, 2019 [13]	1. NGT removal on POD1<br>2. Oral sips on POD1<br>3. Oral liquid diet on POD2<br>4. Urinary catheter removal on POD2<br>5. Epidural catheter removal on POD2<br>6. IV fluid stopped on POD 3<br>7. Drain removed on POD 4<br>8. Tolerating normal diet on POD5<br>9. Discharge on POD6<br>10. Length of stay<br>11. Operative time<br>12. Blood loss<br>13. Readmission & Management<br>14. Delayed gastric emptying<br>15. Postoperative pancreatic fistula<br>16. Post pancreatectomy hemorrhage<br>17. Wound infection<br>18. Burst abdomen<br>19. Dehydration<br>20. Mortality (30-day)
Mangukia et al, 2019 [14]	1. Extubation within 4 hours<br>2. Chest drain removal within 30 hours<br>3. Pneumonia<br>4. Recurrent bronchospasm<br>5. Heart failure<br>6. Pneumothorax

	7. Re-exploration<br>8. Stroke<br>9. Transient rise in creatinine<br>10. Paralytic ileus<br>11. Intestinal obstruction<br>12. Gastrointestinal bleeding<br>13. Sternal wound dehiscence<br>14. Superficial skin infection<br>15. Myocardial infarction<br>16. Mortality (30-day)<br>17. Postoperative length of stay<br>18. Discharge within 100 hours<br>19. Readmission & Management<br>20. Cost<br>21. Patient-satisfaction with care<br>22. Mechanical ventilation time<br>23. re-intubation<br>24. major pulmonary complications<br>25. gastrointestinal complications<br>26. neurological complications<br>27. Persistent atrial fibrillation<br>28. Noninvasive ventilation > 3 times
Nanavati & Prabhakar, 2014 [15]	1. Duration of parenteral analgesia<br>2. Duration of oral analgesia<br>3. Duration of urinary catheter use<br>4. Duration of nasogastric tube use<br>5. Duration of abdominal drain use<br>6. Time to intestinal motility<br>7. Ileus<br>8. Wound infection<br>9. Wound dehiscence<br>10. Anastomotic leakage<br>11. Hospital stay<br>12. Readmission and management
Nanavati & Prabhakar, 2015 [16]	1. Time to ambulation<br>2. Time to removal of all tubes, drains and catheter<br>3. Time to passage of flatus and stool<br>4. Day of discharge

	5. Readmission within 30 days<br>6. Post-operative complications within 30 days<br>7. Anastomotic leak<br>8. Ileus<br>9. Wound infection<br>10. Wound dehiscence<br>11. Operative time<br>12. Blood loss
Pal et al, 2003 [17]	1. Cost<br>2. Use of resources (equipment, medications and investigations)<br>3. Adherence to pathway<br>4. Length of stay<br>5. Operating time<br>6. Bile duct injury<br>7. Missed bile duct stone rate
Pandit et al, 2019 [18]	1) postoperative length of hospital stay<br>2) overall morbidity and major morbidity (Clavien-dindo)<br>3) unplanned reoperation<br>4) 30-day readmission<br>5) mortality<br>6) Post-operative-pancreatic fistula (POPF)<br>7) delayed gastric emptying (DGE)<br>8) post-pancreatectomy hemorrhage (PPH)<br>9) Intrabdominal abscess<br>10) Wound infection<br>11) postoperative ascites<br>12) Chylous ascites
Pillai et al, 2014 [19]	1. Surgical complications<br>2. Mortality<br>3. Delayed gastric emptying<br>4. Pancreatic fistula<br>5. Post pancreatectomy haemorrhage<br>6. Readmission<br>7. Duration of surgery<br>8. Blood loss<br>9. Intraoperative blood transfusion<br>10. Intra-operative fluids

	11. Re-laparotomy<br>12. Length of post-operative hospital stay<br>13. Length of post-operative high dependency ward stay<br>14. Intra-abdominal collection<br>15. Atelectasis of the lung<br>16. Urinary tract infection<br>17. Wound infection<br>18. Nasogastric tube removal<br>19. Oral liquid diet<br>20. Oral solid diet<br>21. Abdominal drain removal<br>22. Passing stool
Pirzada et al, 2017 [20]	1. The time duration of passage of first flatus (hours)<br>2. The time duration of passage of faeces (hours)<br>3. Total length of hospital stay (days)<br>4. Prolonged ileus<br>5. Wound infection
Quader et al, 2010 [21]	1. Mean time to extubation (minutes)<br>2. Post-operative ionotrope requirement<br>3. Length of stay in ICU (hours)<br>4. Re-intubation<br>5. Re-exploration<br>6. Re-admission to ICU<br>7. Length of stay in hospital after surgery (hours)<br>8. Superficial wound infection<br>9. Pleural effusion<br>10. Major complications (in-hospital and out-of-hospital)<br>11. Readmission to hospital<br>12. 30-day mortality
Sahoo et al, 2014 [22]	1. Length of postoperative hospital stay<br>2. Time to passage of first flatus<br>3. Intraoperative and postoperative complications<br>4. Readmission rate<br>5. 30 day mortality<br>6. Serum levels of C-reactive protein<br>7. Operation time<br>8. Time spent in the recovery room

	9. Time to walking<br>10. Time to removal of catheter<br>11. Wound infection<br>12. Cardiac complications<br>13. Pulmonary complications<br>14. Thromboembolic complications<br>15. Urinary tract complications<br>16. Anastomotic leakage<br>17. Bowel obstruction
Sanad et al, 2019 [23]	1. Postoperative length of hospital stay (primary)<br>2. Postoperative pain (VAS)<br>3. Postoperative morbidity: acute confusion, nausea and vomiting, postoperative fever, secondary haemorrhage, atelectasis, DVT, acute urinary retention, paralytic ileus, surgical site infection, urinary tract infection, pneumonia, sepsis or septic shock<br>4. Operative time<br>5. Unplanned return to OR<br>6. Readmission rate due to bowel dysfunction or wound dehiscence or infection<br>7. Blood loss<br>8. Intraoperative fluid use<br>9. Postoperative fluid use<br>10. Adherence to ERAS protocol components
Shah et al, 2016 [24]	1. Pancreatic fistula<br>2. Delayed gastric emptying<br>3. Length of hospital stay<br>4. Readmission rate and treatment<br>5. Mortality<br>6. Bile leakage<br>7. Wound infection<br>8. Operative blood loss<br>9. Blood transfused (units)<br>10. Operative time<br>11. Need for intraoperative blood transfusion<br>12. Use of feeding jejunostomy<br>13. Removal of nasogastric tube on POD1<br>14. Allowing oral liquids POD1<br>15. POD for allowing normal diet<br>16. POD for removing drain<br>17. Days in ICU<br>18. Intra-abdominal abscess

	19. Cardiovascular disease complications<br>20. Respiratory disease complications<br>21. Urinary tract infection<br>22. Thromboembolic disease complications<br>23. Neurological disease complications<br>24. Pneumonia
Shetiwy et al, 2017 [25]	1. PRIMARY: Hospital discharge (day)<br>2. Time to successful enteral feeding (day)<br>3. Time of removal of urinary catheter (day)<br>4. Time of removal of nasogastric tubes (day)<br>5. Time of removal of abdominal drains (day)<br>6. Number of patients undergoing early 'enforced' ambulation (part of pathway)<br>7. Readmissions<br>8. Postoperative nausea and vomiting<br>9. Postoperative ileus<br>10. Anastomotic leak<br>11. Wound infection<br>12. Respiratory tract infection<br>13. Intra-abdominal collection<br>14. Urine retention<br>15. Pulmonary embolism<br>16. Urinary tract infection<br>17. Acute abdomen<br>18. Wound bursting<br>19. Urinary bladder tear<br>20. Ureteric leak<br>21. Intra-hospital mortality
Shrikhande et al, 2013 [26]	1. Post-operative pancreatic anastomotic leak/fistula<br>2. Delayed gastric emptying<br>3. Bile leak<br>4. Post-pancreatectomy hemorrhage<br>5. Mortality<br>6. Hospital stay<br>7. Morbidity
Vashistha et al, 2018 [27]	1. Postoperative complications (Clavien-Dindo)<br>2. 30-day mortality<br>3. 30-day readmission

4. Cost of hospitalization
5. Length of stay
6. Relaparotomy

Table 4: Enablers and Barriers to perioperative care pathway implementation

(The CFIR construct is named with the associated article specific data in brackets. Where appropriate, direct quotations have been used)

Source	Enablers to care pathway implementation	Barriers to care pathway implementation
Agarwal et al, 2018 [1]	1. *Evidence Strength & Quality* ("[ER] was initially studied in colorectal surgery and now encompasses whole range of major gastrointestinal, urological, orthopaedic, gynaecological, and head and neck surgery with improved outcomes like early recovery, shorter hospital stay, and lower rates of complications" and "Whilst ER programme seems successful across various surgeries in the developed nations, very little is known about its feasibility and impact on outcomes in developing nations.")<br>2. *Adaptability* ("Thirteen elements were implemented out of 24 listed in the guidelines published by the ERAS society. This made the implementation process realistic, relatively easier, and successful; however, periodic reinforcements were needed.")<br>3. *Implementation Climate - Tension for Change* ("Over 80% of our patients do not have an insurance cover and spend out of pocket for any hospitalisation expenses. We demonstrated that improved compliance with simple elements in the ER programme was associated with a significant reduction in major complications and postoperative stay; this can have a huge impact on direct and indirect costs to the patients.")<br>4. *Implementation Climate - Compatability* ("Some of the ERAS society recommendations were already in place such as quitting smoking and tobacco before surgery (as a tertiary cancer centre, all patients are counselled for the same), perioperative glycaemic control, and multimodal postoperative analgesia")<br>5. *External Policy & Incentives* ("Over the last decade, ER programme has gained tremendous momentum globally. The UK embraced the concept and developed the ERAS UK around 2010. The USA launched American Society of Enhanced Recovery (ASER) in 2014. There is some evidence from China regarding the feasibility and implementation of ERAS.")<br>6. *Engaging* ("A multidisciplinary team consisting of gastrointestinal surgeons, anaesthetists, trainees, nurse, physiotherapists, and nutritionists was	1. *Cost* ("Some recommendations were not implemented for various reasons such as the following: (a) preoperative immune-nutrition and intraoperative goal-directed fluid therapy were not feasible financially" and "During implementation of ER elements, we faced challenges due to limited resources including manpower and funding coupled with increased volume of referred patients.)<br>2. *Implementation Climate - Compatability* ("Some recommendations were not implemented for various reasons such as the following: (c) being a referral centre, preoperative selective biliary drainage was not feasible, as patients presented to the hospital after the drainage procedure.")<br>3. *Readiness for Implementation - Available Resources* ("During implementation of ER elements, we faced challenges due to limited resources including manpower and funding coupled with increased volume of referred patients"" and ""one of the challenges was rotating surgical trainees and nurse every few months, educating periodically mitigated this problem.")<br>4. *Knowledge & Beliefs about the Intervention* ("Some recommendations were not implemented for various reasons such as the following: (b) surgeons preferred to administer somatostatin analogues to majority of the patients until May 2016 thereafter it was discontinued.")

	trained regarding enhanced recovery elements during the perioperative period" and "Implementation of ER programme involved building a team and circumventing challenges within each discipline.")	
Ahmed et al, 2010 [2]	1. *Evidence Strength & Quality* ("The safety of early extubation practice has been extensively reported in the last 20 years and growing experience shows that early extubation is possible and safe in low risk cardiac surgery cases. International studies report that extubation in fewer than 4 hours may offer accelerated recovery, suggesting that efforts to reduce extubation times further might be worthwhile.")<br>2. *Relative Advantage* ("Adopting a policy of fast tracking is one of the practices greatly helping us in achieving our goal of early recovery and shorter ICU stay" and "Our aim was to determine the applicability and feasibility of fast tracking in adult cardiac surgery at PIMS and to provide all the proposed beneficial effects of this technique to our patients")<br>3. *Peer Pressure* ("Fast tracking in adult cardiac surgery is proving to be successful internationally" and "As soon as cardiac surgery started at our institute, every effort was made to keep this at par with the international standards")	None
Akhtar et al, 2000 [3]	1. *Relative Advantage* ("This [critical care methodology] provides a setting in a hospital to reduce variations in clinical process and has also improved quality of patient care with reduced hospital stay" and "The critical pathway maximises the use of resource. It helps us identify areas of improving not only quality of patient care but also outcome. It is a tool that enables us and the administrators to measure their proficiency and efficiency.")<br>2. *Adaptability* ("if they [patients] were unable to reach by 7:00 A.M. adjustments were made to suit the patient and the operation was scheduled for the afternoon.")<br>3. *Trialability* ("Critical Pathway methodology has been in use for PDA since June 1994 and has now been expanded to surgery for other congenital and acquired heart conditions like Atrial Septal Defects, Pulmonary Artery Banding, Ventricular Septal Defects and Ischaemic Heart Disease")<br>4. *Patient Needs & Resources* ('In our society when a child is admitted for surgery the whole family migrates from the village to the hospital and the cost of boarding and lodging of the family is borne by the parents. So the longer the stay more the cost. We have managed to reduce the operative and ICU costs by efficient management of available resources')<br>5. *Networks & Communications* ("The patient and their parents in case of children were reviewed in the outpatient by the surgeon, cardiac anaesthetist	1. *Cost* (Minimally invasive techniques have been developed to ligate the ductus. All these techniques do in fact reduce the hospital stay, ICU stay and remove the cost of operation theatres. Unfortunately these devices come with a high price tag which is a heavy burden on the economies of the developing countries. We at the Punjab Institute of Cardiology have financial constraints and cannot offer transcatheter closure due to the exuberant cost

	and intensive care sister" and "We have managed to reduce the operative and ICU costs by efficient management of available resources and cooperation of different departments")	
Baluku et al, 2020 [4]	1. *Adaptability* ("We adopted ERAS protocols for gynecologic/oncology surgery and consensus guidelines from the consensus workshop and survey in the United Kingdom, with modifications to our setting and urgency of the surgery.")<br>2. *External Policy & Incentives* ("We adopted ERAS protocols for gynecologic/oncology surgery and consensus guidelines from the consensus workshop and survey in the United Kingdom")	1. *Relative Advantage* ("Despite the potential benefits of ERAS protocols, ERAS has not been tested in emergency situations in low-income settings, and there is uncertainty as to whether the same benefits observed in the HICs will be replicated in resource-limited settings")<br>2. *Cost* (Thromboprophylaxis was not given, nor was ondansetron for postoperative nausea and vomiting (PONV) because the medications are expensive in our low-income setting)
Bansal et al, 2020 [5]	*1. Evidence Strength & Quality* ("ERAS items, as detailed by the ERAS Society, are supported by level one evidence in colorectal surgery for the reduction of complications (~50%) and length of hospital stay (LOS) (~2.5 days).")<br>*2. Relative Advantage* ("Although originally formulated for colorectal surgery, ERAS protocol may be applicable to any major surgery, including RC.")<br>*3. External Policy & Incentives* ("ERAS items, as detailed by the ERAS Society, are supported by level one evidence in colorectal surgery for the reduction of complications (~50%) and length of hospital stay (LOS) (~2.5 days).")	*1. Evidence Strength & Quality* ("acceptance of ERAS protocol by urologists has been slow with main barriers cited as a lack of convincing evidence, disbelief in the ERAS concept, and lack of institutional support.")<br>*2. Implementation Climate - Compatability* ("Implementation in the Indian population poses further unique challenges such as the lack of proper step-down facilities and dedicated nursing staff for patient follow-up after discharge, different dietary patterns, and nonavailibility of some key components of ERAS protocol including carbohydrate-rich drinks and opioid antagonist alvimopan" and "In our center, majority of patients come from a poor socioeconomic background, far off places, and do not have a clean place to stay outside the hospital. Therefore, even if the patient is medically fit for discharge, the patients are kept an extra day or two till they are able to find accommodation and are able to do self-care.")<br>*3. Readiness for Implementation - Available Resources* ("We could not include the use of carbohydrate-rich clear drinks and alvimopan in the current study due to nonavailability")<br>*4. Knowledge & Beliefs about the Intervention* ("acceptance of ERAS protocol by urologists has been slow with main barriers cited as a lack of convincing evidence, disbelief in the ERAS concept, and lack of institutional support.")
Chaudhary et al, 2015 [6]	*1. Evidence Strength & Quality* ("Reduction in hospital stay after PD has been reported in few recent publications that incorporated ERAS protocols in the postoperative management")<br>*2. Adaptability* ("Strategies that can be implemented as part of the ERAS protocol (for future analysis) include preoperative and postoperative patient education, monitoring the depth of anesthesia (to titrate the anesthetic agents and myorelaxants), and postoperative lung recruitment maneuvers.")	None

	3. External Policy & Incentives ("Enhanced recovery after surgery (ERAS) protocols, as an integral part of perioperative patient care, have been readily embraced by colorectal surgeons with reports of its application in other specialties such as vascular surgery, head and neck surgery, and urology.<br>*4. Networks & Communications* ("All surgeries were performed by a single surgeon with considerable experience in pancreatic surgery.")	
Iyer & Kareem, 2019 [7]	*1. Evidence Strength & Quality* ("There have been many studies on the effect of FTP on colorectal surgeries[3] as well as colostomy closures.[4]")<br>*2. External Policy & Incentives* ("FTPs or enhanced recovery programs are treatment protocols made with adherence to evidence-based principles.")	*1. Patient Needs & Resources* ("In our study, inadequate pain relief and distance from the hospital to the patient's residence were the major factors that prevented the patients from being discharged on time, thus increasing duration of hospital stay, as well as reducing the patient turnover for the hospital.")<br>*2. Knowledge & Beliefs about the Intervention* ("Although the ERAS protocol has been in practice in various countries and hospitals, it is still not very widely followed in our country. This is due to many surgeons preferring to opt for traditional methods, rather than adopting the new evidence-based protocols.")
Jain et al, 2015 [8]	*1. Cost* ("Most of our patients do not have health insurance and have to pay for their implants directly to the hospitals. These factors make a cemented bipolar prosthesis a good choice for our patients.")<br>*2. Patient Needs & Resources* ("We have developed a comprehensive geriatric hip fracture program in order to improve the results of patients with these injuries keeping in mind the specific features and demands of our patient population." and "The cemented modular bipolar arthroplasty ... helps to fulfill the functional demands and expectations of the elderly Indian population. It is cost-efficient which is of major importance to our patients.")<br>*3. Cosmopolitanism* (Coauthor: Orthopaedic Surgeon, Baylor Regional Medical Center, Plano, TX, USA, and “The program was modeled after the hip fracture program at the University of Rochester.")<br>*4. Networks & Communication* ("The Departments of Orthopedics and Inter- nal Medicine worked together to formulate a treatment algorithm for geriatric hip fractures for our institution ... The Departments of Anesthesia, Physical therapy, and Nursing were sensitized to the special needs and protocols of this program")<br>*5. Implementation Climate - Compatability* ("95% were sent home within 14 days after surgery with the length of stay being shorter as compared to other Indian studies. This is due to comprehensive geriatric care under our program and the good social support structure at home existing in our region")<br>*6. Engaging* (The Departments of Orthopedics and Inter- nal Medicine	*1. Cost* ("There is a lack of good-quality cementless stems or THA systems manufactured locally at cheaper costs. Most of our patients do not have health insurance and have to pay for their implants directly to the hospitals.")<br>*2. Knowledge & Beliefs About the Intervention* ("The anesthesia team advised surgical delay for patients on clopidogrel after 5 days of last ingestion of tablet in view of the potential for bleeding" and "Recent studies have shown that early surgery in patients on clopidogrel and warfarin is safe with no increase in observed complications.")

	worked together to formulate a treatment algorithm for geriatric hip fractures for our institution ... The Departments of Anesthesia, Physical therapy, and Nursing were sensitized to the special needs and protocols of this program")	
Khowaja, 2006 [9]	1. *Relative Advantage* ("The literature outlines several benefits of clinical pathways" - the narrative provides several examples from the literature and "The delivery of nursing care to patients therefore becomes a process of thinking as well as doing, as nurses continuously monitor variances in the use of clinical pathways and work to prevent such variances recurring by monitoring patient outcomes in contrast to the traditional approach to the nursing process.")<br>2. *Knowledge & Beliefs about the Intervention* ("Clinical pathways use a multidisciplinary approach to the delivery of patient care; therefore the researcher saw the value of testing this concept of patient care in her work setting and, in the event of supportive findings, changing the model of nursing practice at Aga Khan University Hospital (AKUH) from the traditional to the multidisciplinary approach")<br>3. *Reflecting & Evaluating* ("Considering the size of the project and the number of variables required to be measured, the data collection plan was thoroughly outlined, and all suggestions outlined during pilot phase of study were rigorously followed to ensure proper collection, recording and storing of data")<br>4. *Engaging - Champions* ('The researcher also had ongoing dialogue with AKUH unit staff to explore any ambiguities in design and content of the clinical pathway and to ensure the successful implementation of the pathway")<br>5. *Planning* (utilising the King's interacting systems framework and theory of goal attainment)	1. *Cost* ("Clinical pathways can be applied to all specialty areas however constraints such as time, budget, and the size of the project, as well as the researcher's involvement, led the researcher to narrow the scope of the study to one disease and one discipline")<br><br>2. *Readiness for Implementation - Available Resources* ("Clinical pathways can be applied to all specialty areas however constraints such as time, budget, and the size of the project, as well as the researcher,Äôs involvement, led the researcher to narrow the scope of the study to one disease and one discipline")<br><br>3. *Reflecting & Evaluating* ("The final limitation of the study was its inability to communicate the analysis of variances and feedback to the multidisciplinary team, which is a vital component of the entire clinical pathway program. The study did not include this component in the design where the results of variances could be shared with all stakeholders, particularly physicians and nurses, and practices modified to further improve them. This approach was omitted to prevent the occurrences of biases by the researcher"
Kulshrestha et al, 2019 [10]	*1. Peer Pressure* ("Expeditious surgical care of geriatric hip fractures is gradually being accepted as the standard of care at most centers around the world."")<br>*2. External Policy & Incentives* ("Expeditious surgery (within 24 hours) after hip fracture in the elderly patients is considered the standard of care in the current treatment guidelines of Western countries")<br>*3. Networks & Communication* ("A dedicated multidisciplinary team of physicians-surgeons, anesthetists, nursing coordinators and physio-occupational therapists—ran the GHFP.")<br>*4. Readiness for Implementation - Available Resources* ("As a military hospital with adequate resources, we were able to institute the GHFP, which	*1. Evidence Strength & Quality* ("there is conflicting literature wherein some studies have shown patient optimization before surgery is a more important determinant of early mortality rate than time to surgery is" and "Time to surgery still remains a contentious factor; some studies categorically showed that when the effects of other confounders were controlled, time to surgery had insignificant effect on mortality after hip fracture surgery.")<br>*2. Implementation Climate - Compatability* ("In developing countries, because health care facilities are not available or unaffordable, the comor-bidities are inadequately managed. Usually, these patients have delayed presentation (24–72 hours after injury) with missed meals and medications." and "in developing countries, like India, expeditious surgery is al- most

	include all these aspects of comprehensive geriatric hip fracture care"" and "these goals could be met in our patients because they were treated in the military facility with availability of in-hospital rehabilitation beds.")<br>5. *Planning* ("An orthopaedic matron (PS) was designated as the dedicated trauma coordinator for fast tracking patients with hip fractures to surgery." and "We initiated the Geriatric Hip Fracture Registry, in which data that would help us study the effect of the program was recorded.")	impossible because of the limited presence of healthcare facilities and poor socioeconomic conditions.")
Kurmi et al, 2020 [11]	1. *Evidence Strength & Quality* ("Enhanced Recovery after Surgery (ERAS) is an evidence-based protocol designed to standardize medical care, improve outcomes, and lower health care costs"<br>2. *Relative Advantage* ("With implementation of these elements, the postoperative outcome in patients undergoing stoma closure may be improved.")<br>3. *External Policy & Incentives* (Enhanced Recovery after Surgery (ERAS)	None
Kuzmenko et al, 2019 [12]	1. *Evidence Strength & Quality* ("Most authors agree on a positive impact ERAS program on early discharge of patients after PD"<br>2. *Relative Advantage* ("The use of the ERAS program with PD significantly reduces the number of postoperative complications, pro- motes earlier mobilization of patients, their early return to physiological nutrition, and reliably reduces LoS")<br>3. *External Policy & Incentives* ("In 2012, the ERAS organization established guidelines for the first-line management of patients with PD.")	1.*Complexity* (full implementation of the ERAS program is not always possible)
Mahendran et al, 2019 [13]	*1. Evidence Strength & Quality & Relative Advantage* ("The average length of hospital stay (LOS) after colorectal surgery has decreased to 4 days by following ERAS protocol. Similar promising studies have been reported after gynecological, breast, liver, urological, gastric and bariatric surgeries." and "Few recent systematic reviews and meta-analysis have found ERAS protocol to be beneficial for patients undergoing PD")<br>*2. Relative Advantage* ("ERAS protocol is an evidence-based approach to reduce surgical stress and enhance recovery in the postoperative period.")	*1. Evidence Strength & Quality* ("only limited studies are available worldwide regarding application of the ERAS protocol after pancreaticoduodenectomy (PD)" and "A PubMed search revealed only two studies from India on ERAS after PD")<br>*2. Knowledge & Beliefs about the Intervention* ("many surgeons prefer a conservative approach in the postoperative period after PD" and "Because of the fatal major postoperative complications, surgeons prefer a more conservative approach that leads to increase in LOS")
Mangukia et al, 2019 [14]	*1. Evidence Strength & Quality* ("A safe fast-track (FT) cardiac anesthesia is well established" and "In western countries, FT and ultrafast-track cardiac surgery has already been studied. It was also found that FT coronary artery bypass grafting" and "is practical and realistic even in rural areas.")<br>*2. Relative Advantage* ("We combined the advantage of OPCAB with that of FT surgery to extract the maximum benefit within safety limits")<br>*3. Adaptability* ("As our experience with FT grew, we started using	*1. Evidence Strength & Quality* ("FT OPCAB has never been studied in the Indian population")

	benzodiazepines on day 1 more generously as a sleep aid as well as for anxiety.")<br>4. External Policy & incentives ("A safe fast-track (FT) cardiac anesthesia is well established" and "In western countries, FT and ultrafast- track cardiac surgery has already been studied. It was also found that FT coronary artery bypass grafting")<br>*5. Networks & Communication* ("The FT team included cardiac anesthetists, physicians, surgeons, intensivists, and physiotherapists")<br>*6. Engaging* (The FT team included cardiac anesthetists, physicians, surgeons, intensivists, and physiotherapists)	
Nanavati & Prabhakar, 2014 [15]	*1. Evidence Strength & Quality* ("Many review articles have been written, and meta-analyses and clinical trials have been performed in this field." and "Several studies and meta-analyses give adequate evidence that these measures are appropriate when applied independently or as part of an enhanced recovery program")<br>*2. Adaptability* ("It is not a fixed protocol that can be applied to any clinical setting. It rather provides certain basic principles on the basis of which a protocol that best suits the patient, clinical team and institution can be formulated." and "In this study, we delineated a protocol that suited our institution and infrastructure.")<br>*3. Relative Advantage* ("This study shows that the ability to apply fast-track surgery is not governed by the capability of patient or hospital to spend money. It rather turns out to be extremely cost-efficient, saving both the hospital and patient ample money and resources." and "An earlier discharge has a lot of advantages: it saves considerable amount of healthcare costs to the patient, pro- motes a sense of well-being in the patient and allows for early re-integration of the patient into the society. At an institutional level, it increases patient turnover and reduces expenses of the hospital. The latter is very important in a country like India where public sector hospitals are the majority stake holders in healthcare.")<br>*4. Peer Pressure* ("The principles of fast-track surgery are gradually being adopted in all surgical specialties.")	*1. Evidence Strength & Quality* ("Because there is a significant variability in the components of each fast-track program, results are difficult to compare. Unfortunately, there is a paucity of large randomised prospective trials")<br>*2. Implementation Climate - Compatability* (""The Indian population usually comes from an underprivileged class. This has direct bearing on the patient's nutritional status and co- morbid features which may have increased the risk of anastomotic dehiscence and peri-operative complications. Due to financial constraints of the patients and restricted facilities at our government hospital, it was not possible to perform assessment of serum markers like pre-albumin and C-reactive protein, but rather we had to use markers like albumin which were available to us." and "A problem that is faced by such enhanced recovery program is that since they include several components that depart from traditional knowledge, it is not accepted across the board with all surgeons. There is a lot of inertia in certain surgeons to try out newer methods even though they may have evidence to support their use.")<br>*3. Readiness for Implementation - Available Resources* ("Due to financial constraints of the patients and restricted facilities at our government hospital, it was not possible to perform assessment of serum markers like pre-albumin and C-reactive protein, but rather we had to use markers like albumin which were available to us.")<br>*4. Knowledge & Beliefs about the Intervention* ("A problem that is faced by such enhanced recovery program is that since they include several components that depart from traditional knowledge, it is not accepted across the board with all surgeons. There is a lot of inertia in certain surgeons to try out newer methods even though they may have evidence to support their use")
Nanavati & Prabhakar, 2015 [16]	*1. Evidence Strength & Quality* ("Multi-modal interventions implemented in the peri-operative period have been known to enhance recovery." and "There	*1. Cost* ("We did not have the technology available at other leading FTS centres like transesophageal Doppler, etc., to monitor GDFT [goal directed

	is sufficient evidence to suggest that the multiple interventions in a FTS protocol have a positive impact on bowel function" and "It has been conclusively proven that the simultaneous application of all the individual components ofFTS yield maximum advantage")<br>2. *Adaptability* ("We therefore tailored a protocol that would work best with the limited facilities available at our centre. It has been shown that FTS need not be a rigid protocol but can be flexible to suit individual patients and local institutional needs")<br>3. *Patient Needs & Resources* ("when a patient comes in for a colostomy reversal he/she does not expect a repeat of the "major" surgery they had undergone to land up with a stoma in the first place. They expect a smooth peri-operative course and earlier return to productivity. It is because of the above reasons (especially the latter) that it might be in the interest of the surgeon to apply the FTS principles to colostomy closures")<br>4. *External Policy & Incentives* ("Starting from colorectal surgery, this methodology has gradually been adopted by various sub-specialties")<br>5. *Knowledge & Beliefs about the Intervention* ("We believe that outcome may be significantly improved with multimodal interventions in the peri-operative care of patients undergoing this procedure.")	fluid therapy]. The costs involved have been prohibitive at governmental organization like ours.")<br>*2. Evidence Strength & Quality and Intervention Source* ("Studies dealing with FTS in colorectal surgery in the West have traditionally involved surgeries performed predominant- ly for malignancy. In a country like India, infectious and inflammatory bowel diseases still account for the majority of colorectal surgery." and "There have been very few reports studying the effect of FTS on colostomy closures.")<br>*3. Readiness for Implementation - Available Resources* (However, in countries like India, we find it difficult to implement a few components of the program. We therefore tailored a protocol that would work best with the limited facilities available at our centre." and "We did not have the technology available at other leading FTS centres like transesophageal Doppler, etc., to monitor GDFT [goal directed fluid therapy]. The costs involved have been prohibitive at governmental organization like ours.")
Pal et al, 2003 [17]	1. *Relative Advantage* ("An analysis of these 6 categories revealed a number of areas in which changes in practice could be introduced as part of a clinical pathway, with the aim of reducing costs and variability of practice without compromising quality of care")<br>2. *Implementation Climate - Goals & Feedback* ("Recommendations addressing changes in the process of care were developed from this review and presented to the general surgery group, which is made up of consultants, residents, and nursing staff. Consensus for change was reached, and the recommendations were used to develop and introduce a clinical pathway for laparoscopic cholecystectomy")<br>3. *Planning* ("Recommendations addressing changes in the process of care were developed from this review and presented to the general surgery group, which is made up of consultants, residents, and nursing staff. Consensus for change was reached, and the recommendations were used to develop and introduce a clinical pathway for laparoscopic cholecystectomy")	*1. Networks & Communications* ("We felt that the advantages were more relevant for day care procedures and discussed the issue with the anesthesia department. No firm commitment was made to begin a change in practice...")
Pandit et al, 2019 [18]	1. *Evidence strength and quality* (With implementation of ERAS protocol, several systemic review and meta-analysis has demonstrated its safety, with shortened postoperative hospital stay, reduced overall morbidity without affecting readmission and mortality rates)	1. *Relative advantage* ("its implementation has lagged for pancreatic surgeries. It was not until 2007, where the ERAS were first implemented in a pancreatic surgery, probably due to fear of increase morbidity and mortality, because of its greater complexity" AND "due to the safety concerns,

	2. *Relative Advantage* ("the guidelines for ERAS in pancreatic surgery were published in 2012 by the ERAS society, following which an array of studies has shown an excellent outcome")<br>3. *External Policy & Incentives* (the ERAS guidelines for pancreatic surgery 2012)	application of ERAS program in the perioperative period of pancreatic surgery is still being explored in centres of Nepal")
Pillai et al, 2014 [19]	*1. Evidence Strength & Quality* ("Several studies have demonstrated the effectiveness of this programme in colonic resection. Recently, fast-track surgery has been attempted in pancreatic surgery with encouraging results, but such data are sparse")<br>*2. Cost* ("This protocol is practical and can be easily introduced with no increase in cost.")	None
Pirzada et al, 2017 [20]	1. *Evidence Strength & Quality* (discusses meta-analyses comparing enhanced recovery after surgery (ERAS) with conventional methods and "The promising results of the fast-track programmes in bowel surgeries led to other surgical specialties to incorporate and apply fast-track protocols in their surgeries and they too showed promising results.")<br>2. *Relative Advantage* ("Adopting ERAS protocol would improve peri-operative management and benefit patients" and "[implementation of ERAS] translates into lower days of hospital bed occupancy, hence reduces cost to the government and the load on the public hospitals.")<br>3. *Adaptability* ("The ERAS protocol has been modified in different studies according to the availability of resources and has been applied in different surgeries." and this study also used a modified version of the ERAS protocol)<br>4. *Trialability* ("We also tried to establish a definitive role of the ERAS protocol in decreasing the length of stay and early resolution of ileus in patients undergoing stoma reversal so that the better of the two protocols would be used in future")<br>5. *External Policy & Incentives* ("Adopting ERAS protocol")<br>6. *Tension for change* ("The healthcare system of Pakistan is underfunded and overstretched and these additional cases inevitably put additional burden on the already limited hospital beds and resources." and therefore a driver for implementation for a program that "reduces cost to the government and the load on the public hospitals.")	None
Quader et al, 2010 [21]	1. *Evidence Strength & Quality* ("Fast-track recovery protocols gained worldwide popularity and have contributed to significant reductions in the postoperative hospital stay and cost without any increase in postoperative mortality and morbidity" and "Multiple studies from the Western world have	1. *Cosmopolitanism* ("There is also lack of facilities for regular follow up of the patient outside the operating hospital which causes hesitation on the part of the operating surgeon to discharge the patient early." and "All patients in the fast track group were required to have an accompanying family member

	shown the safety and efficacy of fast-track cardiac surgery.")<br><br>2. *Relative Advantage* ("significant reductions in the postoperative hospital stay and cost without any increase in postoperative mortality and morbidity")<br><br>3. *Tension for change* (We need to get used to fast-track pathway to make full use of a limited health care resource)	or a friend available for hospitalization time and for 72 hours thereafter and they should have accommodation to stay in Dhaka city for at least 1 week after discharge.")<br>2. *Implementation Climate - Compatibility* ("Though it is well established that most patients with minimal co-morbid conditions and stable haemodynamics can be safely admitted on the day of surgery, here patients stays for many days preoperatively... as they are admitted without complete workup, which is performed once the patient is in-hospital")<br>3. *Knowledge & Beliefs about the Intervention* ("some case patient wants to stay in hospital as they are apprehensive of any complication that may arise after discharge." and "The poor educational level of patients / parents is another factor that causes a sense of insecurity if the patient is discharged early from the hospital." and "lack of a set protocol and good understanding between the members of cardiac surgical team about fast- tracking the patients")
Sahoo et al, 2014 [22]	*1. Evidence Strength & Quality* ("The results that can be achieved with ERAS(FTS)-reductions in postoperative morbity, average length of hospital stay and the consumption of resources-are, however, significant.")<br>*2. Relative Advantage* ("So this ERAS is advanced perioperative care for better outcome of the patient.")<br>*3. Adaptability* ("The FTS protocol was developed by us which can fit to our setup by bringing few modifications to the published protocols.")<br>*4. Knowledge & Beliefs about the Intervention* ("The results that can be achieved with ERAS(FTS)-reductions in postoperative morbity, average length of hospital stay and the consumption of resources-are, however, significant.")	None
Sanad et al, 2019 [23]	1. *Relative Advantage* ("Implementation of ERAS protocol is linked with the decreased hospital live length, a reduction in rates of postoperative difficulty, reduced illness, and cost funds, while conserving patient approval and life quality[1,2]")<br>2. *External Policy & Incentives* (ERAS protocols)<br>3. *Readiness for Implementation - Available Resources* ("The current study was done and funded by Minia Maternity University Hospital as Scientific Research Plan")<br>4. *Engaging - Champions* ("In our opinion, the most decisive point was to operate a devoted nurse to supervise the procedure from admission till discharge")	1. *Networks & Communications* ("One of the most important difficulties we faced during implementation of such a program is the need of joint effort between various wellbeing experts and various strengths.")<br><br>2. *Readiness for Implementation - Available Resources* ("unavailability of laparoscopic hysterectomy of endometrial carcinoma in our hospital")

Shah et al, 2016 [24]	*1. Evidence Strength & Quality* ("Encouraging results with fast track programs after PD have been reported elsewhere" and "Fast track surgery is to improve the postoperative outcome by reducing hospital stay and readmission rate.")<br>*2. Structural Characteristics* ("a regional center of reference for pancreatic surgery. Our single team at this center introduced fast track strategy for PD soon after acquiring adequate skills and experience in the area and evolved a focused approach to achieve its objectives" and "The volume of PD increased from an average of 15 per year in the conventional group to around 28 per year in the fast track group, indicating the presence of a learning curve.")<br>*3. Readiness for Implementation* ("a regional center of reference for pancreatic surgery. Our single team at this center introduced fast track strategy for PD soon after acquiring adequate skills and experience in the area and evolved a focused approach to achieve its objectives")<br>*4. Networks & Communication* ("treated between June 2008 and September 2012 by a single team headed by a senior pancreatic surgeon")<br>*5. Engaging* ("Analgesia requirements were monitored in both groups by a dedicated anesthetic pain service team.")	None
Shetiwy et al, 2017 [25]	1. *Relative Advantage* ("In order to handle the metabolic response of surgery and speed up the recovery of patients, several enhanced recovery protocols have been formulated with encouraging results. Reduced incidence of postoperative morbidity and mortality, shorter hospital stay, as well as reduction in healthcare costs were among these results")<br>2. *Adaptability* ("Different versions of the enhanced recovery protocols have been established by combining various sets of elements, and no set is considered an ideal protocol of enhanced recovery; thus, authors all around the world have been utilizing a series of programs that focus on specific key components to improve the recovery pathway. This study was designed to implement the concept of fast track care and to set a policy of enhanced recovery after surgery protocols into the surgical management of patients with a colorectal carcinoma at our oncology center" and "Fifteen items were formulated into an enhanced recovery protocol in our study" and "Although meta-analyses have reported no value of the routine use of drains for colorectal surgeries regarding the postoperative morbidities, we found that they were useful in detecting anastomotic leaks.")	None
Shrikhande et al, 2013 [26]	*1. Structural characteristics* ("Retrospectively and prospectively maintained databases of the Gastrointestinal and Hepato-Pancreato-Biliary (GI and HPB)	*1. Structural Characteristics* ("Other contributory reasons could be infrastructure and resource constraints as evidenced by poorly developed

	Surgical Oncology Unit were analyzed" and "Data from developed countries shows that overall mortality is higher following PD in low-volume hospitals compared to high-volume hospitals but data from developing countries is lacking" and "Additionally, there exists a learning curve for a complex procedure such as PD. Thus, high-volume institutes also provide a good opportunity to impart training to aspiring pancreatic surgeons")<br>2. *Readiness for Implementation* ("In period B, a change in the surgical team led to a thrust toward standardization of the procedure of PD")<br>3. *Networks & Communication* ("Most notable amongst these changes was the constitution of the Gastrointestinal Disease Management Group (GI-DMG) from period B onwards. The group is composed of GI and HPB Surgical oncologists, Medical Gastroenterologists, Radiologists, Intensivists, Interventional Radiologists, Radiation and medical oncologists, and dedicated GI pathologists and clinical co-ordinators who are responsible for carefully monitoring the patient's preoperative investigations thereby formulating a holistic plan for every patient")<br>4. *Reflecting & Evaluating* ("Retrospectively and prospectively maintained databases of the Gastrointestinal and Hepato-Pancreato-Biliary (GI and HPB) Surgical Oncology Unit were analyzed")	primary healthcare systems with its negative impact on referral patterns, and the scarcity of well developed, tertiary care, multidisciplinary centers")
Vashistha et al, 2018 [27]	1. *Relative Advantage* ("by providing best available resources for those who need them the most, LMIC can achieve emergency laparotomy outcomes comparable to well-endowed Western centers")<br>2. *Adaptability* ("Our EL care protocol was evidence based and kept in perspective the prevalent ground realities in LMIC. Thus, the patients were managed in SICU by a multidisciplinary team well versed in intricacies of modern critical care. This effectively circumvented the likely deficiencies in terms of trained manpower and monitoring equipment, often prevalent in wards.")<br>3. *Evidence Strength & Quality* ("Our EL care protocol was evidence based and kept in perspective the prevalent ground realities in LMIC")<br>4. *Patient Needs & Resources* ("Admittedly the patients presenting to tertiary level private healthcare facility probably have better socioeconomic and perhaps better nutritional status than those visiting public sector hospitals.")<br>5. *Readiness for Implementation - Available Resources* ("An important aspect was that EL was performed by the same team of senior surgical gastroenterologists. This aspect became especially important when dealing with conditions such as emergency presentation of GI tract cancers, GI bleed or acute ischemia that necessitate major resective procedures" and "We appreciate that being a tertiary care hospital some of the usual infrastructural	1. *Intervention Source* ("Most of the available data on the subject have come in from centers in Europe and North America.")<br>2. *Implementation Climate - Relative Priority* ("With focus on primary/preventive healthcare, surgical diseases are not regarded as priority. Also in a vast majority of LMIC countries including India, state spending on public healthcare is inadequate.")

constraints in LMIC may not have been applicable in our setting.")
6. Structural Characteristics ("This was a new team, in a new hospital with a new protocol" and "We appreciate that being a tertiary care hospital some of the usual infrastructural constraints in LMIC may not have been applicable in our setting.")

References

1. Agarwal V, Thomas MJ, Joshi R, et al (2018) Improved Outcomes in 394 Pancreatic Cancer Resections: the Impact of Enhanced Recovery Pathway. J Gastrointest Surg 22:1732–1742

2. Ahmed N, Khan F, Zahoor M, et al (2010) Fast tracking in adult cardiac surgery at Pakistan Institute of Medical Sciences. J Ayub Med Coll Abbottabad 22:28–31

3. Akhtar RP, Hameed K, Sarwar M, et al (2000) Critical pathway, Cardiac surgery in the third world. Pak J Med Sci Q 16:263–266

4. Baluku M, Bajunirwe F, Ngonzi J, et al (2020) A Randomized Controlled Trial of Enhanced Recovery After Surgery Versus Standard of Care Recovery for Emergency Cesarean Deliveries at Mbarara Hospital, Uganda. Anesth Analg 130:769–776

5. Bansal D, Nayak B, Singh P, et al (2020) Randomized controlled trial to compare outcomes with and without the enhanced recovery after surgery protocol in patients undergoing radical cystectomy. Indian J Urol 36:95–100

6. Chaudhary A, Barreto SG, Talole SD, et al (2015) Early discharge after pancreatoduodenectomy: what helps and what prevents? Pancreas 44:273–278

7. Iyer SP, Kareem Z (2019) Fast-track Protocol versus Conventional Protocol on Patient Outcome: A Randomized Clinical Trial. Niger J Surg 25:36–41

8. Jain D, Sidhu GS, Selhi HS, et al (2015) Early Results of a Geriatric Hip Fracture Program in India for Femoral Neck Fracture. Geriatr Orthop Surg Rehabil 6:42–46

9. Khowaja K (2006) Utilization of King's interacting systems framework and theory of goal attainment with new multidisciplinary model: clinical pathway. Aust J Adv Nurs 24:44–50

10. Kulshrestha V, Sood M, Kumar S, et al (2019) Outcomes of Fast-Track Multidisciplinary Care of Hip Fractures in Veterans: A Geriatric Hip Fracture Program Report. Clin Orthop Surg 11:388–395

11. Kurmi S, Pandit N, Sah S, et al (2020) Safety and Feasibility of Enhanced Recovery after Surgery (ERAS) Protocol in Patients Undergoing Stoma Closure. Indian J Surg 83:703–707

12. Kuzmenko V, Usenko A, Skums A, et al (2019) Perioperative multimodal program of enhanced recovery following pancreaticoduodenectomy. Georgian Med News 290:7–12

13. Mahendran R, Tewari M, Dixit VK, Shukla HS (2019) Enhanced recovery after surgery protocol enhances early postoperative recovery after

pancreaticoduodenectomy. Hepatobiliary Pancreat Dis Int 18:188–193

14. Mangukia C, Kachhadia M, Meswani M (2019) Fast-track off-pump coronary artery bypass: single-center experience. Asian Cardiovasc Thorac Ann 27:256–264
15. Nanavati AJ, Prabhakar S (2014) A comparative study of “fast-track” versus traditional peri-operative care protocols in gastrointestinal surgeries. J Gastrointest Surg 18:757–767
16. Nanavati AJ, Prabhakar S (2015) Fast-Tracking Colostomy Closures. Indian J Surg 77:1148–1153
17. Pal KMI, Ahmed M (2003) Itemized bill: Novel method to audit the process of laparoscopic cholecystectomy. World J Surg 27:666–670
18. Pandit N, Sah R, Awale L, et al (2019) Outcome of Enhanced Recovery after Surgery Protocol in a Patients Undergoing Pancreatic Surgery. JOP 20:4–7
19. Pillai SA, Palaniappan R, Pichaimuthu A, et al (2014) Feasibility of implementing fast-track surgery in pancreaticoduodenectomy with pancreaticogastrostomy for reconstruction--a prospective cohort study with historical control. Int J Surg 12:1005–1009
20. Pirzada MT, Naseer F, Haider R, et al (2017) Enhanced recovery after surgery (ERAS) protocol in stoma reversals. J Pak Med Assoc 67:1674–1678
21. Quader SA, Sarker R, Ahmed F, et al (2010) Fast-track Cardiac Surgery in Children: Feasibility in Bangladeshi Setting. Cardiovascular Journal 3:50–54
22. Sahoo MR, Gowda MS, Kumar AT (2014) Early rehabilitation after surgery program versus conventional care during perioperative period in patients undergoing laparoscopic assisted total gastrectomy. J Minim Access Surg 10:132–138
23. Sanad AS, El-Gindi E, El-Khateeb RR, et al (2019) Implementation of enhanced recovery after surgery for endometrial carcinoma: A non-randomized controlled trial. Indian Journal of Public Health Research and Development 10:1979–1984
24. Shah OJ, Bangri SA, Singh M, et al (2016) Impact of centralization of pancreaticoduodenectomy coupled with fast track recovery protocol: a comparative study from India. Hepatobiliary Pancreat Dis Int 15:546–552
25. Shetiwy M, Fady T, Shahatto F, Setit A (2017) Standardizing the Protocols for Enhanced Recovery From Colorectal Cancer Surgery: Are We a Step Closer to Ideal Recovery? Ann Coloproctol 33:86–92
26. Shrikhande SV, Barreto SG, Somashekar BA, et al (2013) Evolution of pancreatoduodenectomy in a tertiary cancer center in India: improved results from service reconfiguration. Pancreatology 13:63–71
27. Vashistha N, Singhal D, Budhiraja S, et al (2018) Outcomes of Emergency Laparotomy (EL) Care Protocol at Tertiary Care Center from Low–Middle-Income Country (LMIC). World J Surg 42:1278–1284
